# Pharmacological induction of membrane lipid poly-unsaturation sensitizes melanoma to ROS inducers and overcomes acquired resistance to targeted therapy

**DOI:** 10.1186/s13046-023-02664-7

**Published:** 2023-04-19

**Authors:** Ali Talebi, Vincent de Laat, Xander Spotbeen, Jonas Dehairs, Florian Rambow, Aljosja Rogiers, Frank Vanderhoydonc, Lara Rizotto, Mélanie Planque, Ginevra Doglioni, Sahar Motamedi, David Nittner, Tania Roskams, Patrizia Agostinis, Oliver Bechter, Veerle Boecxstaens, Marjan Garmyn, Marie O’Farrell, Alan Wagman, George Kemble, Eleonora Leucci, Sarah-Maria Fendt, Jean-Christophe Marine, Johannes V. Swinnen

**Affiliations:** 1 Department of Oncology, Laboratory of Lipid Metabolism and Cancer, LKI, KU Leuven, 3000 Leuven, Belgium; 2 Department of Applied Computational Cancer Research, Institute for AI in Medicine (IKIM), University Hospital Essen, Essen, Germany; 3 University of Duisburg-Essen, Essen, Germany; 4 Laboratory for Molecular Cancer Biology, VIB Center for Cancer Biology, 3000 Leuven, Belgium; 5 Department of Oncology, Laboratory for Molecular Cancer Biology, KU Leuven, 3000 Leuven, Belgium; 6 Department of Oncology, Laboratory for RNA Cancer Biology, LKI, KU Leuven, Leuven, Belgium; 7 Department of Oncology, Trace PDX Platform, LKI, KU Leuven, Leuven, Belgium; 8 Laboratory of Cellular Metabolism and Metabolic Regulation, VIB Center for Cancer Biology, 3000 Leuven, Belgium; 9 Department of Oncology, Laboratory of Cellular Metabolism and Metabolic Regulation, LKI, KU Leuven, 3000 Leuven, Belgium; 10 Histopathology Expertise Center, VIB-KU Leuven Center for Cancer Biology, 3000 Leuven, Belgium; 11 Department of Imaging and Pathology, KU Leuven and University Hospitals Leuven, Leuven, Belgium; 12 Department of Cellular and Molecular Medicine, VIB-KU Leuven Center for Cancer Biology, KU Leuven, Leuven, Belgium; 13 LKI, Department of General Medical Oncology, Department of Oncology, University Hospitals Leuven, KU, Leuven, Belgium; 14 Department of Oncology, KU Leuven and Department of Surgical Oncology, UZ Leuven, Leuven, Belgium; 15 Department of Oncology and Dermatology, Laboratory of Dermatology, University Hospitals Leuven, University of Leuven, Leuven, Belgium; 16 Sagimet Biosciences, 155 Bovet Rd, San Mateo, CA 94402 USA; 17 3-V Biosciences, Inc, 3715 Haven Ave, Menlo Park, CA 94025 USA

**Keywords:** Lipid metabolism, Therapy resistance, Melanoma

## Abstract

**Background:**

One of the key limitations of targeted cancer therapies is the rapid onset of therapy resistance. Taking BRAF-mutant melanoma as paradigm, we previously identified the lipogenic regulator SREBP-1 as a central mediator of resistance to MAPK-targeted therapy. Reasoning that lipogenesis-mediated alterations in membrane lipid poly-unsaturation lie at the basis of therapy resistance, we targeted fatty acid synthase (FASN) as key player in this pathway to evoke an exquisite vulnerability to clinical inducers of reactive oxygen species (ROS), thereby rationalizing a novel clinically actionable combination therapy to overcome therapy resistance.

**Methods:**

Using gene expression analysis and mass spectrometry-based lipidomics of BRAF-mutant melanoma cell lines, melanoma PDX and clinical data sets, we explored the association of FASN expression with membrane lipid poly-unsaturation and therapy-resistance. Next, we treated therapy-resistant models with a preclinical FASN inhibitor TVB-3664 and a panel of ROS inducers and performed ROS analysis, lipid peroxidation tests and real-time cell proliferation assays. Finally, we explored the combination of MAPK inhibitors, TVB-3664 and arsenic trioxide (ATO, as a clinically used ROS-inducer) in Mel006 BRAF mutant PDX as a gold model of therapy resistance and assessed the effect on tumor growth, survival and systemic toxicity.

**Results:**

We found that FASN expression is consistently increased upon the onset of therapy resistance in clinical melanoma samples, in cell lines and in Mel006 PDX and is associated with decreased lipid poly-unsaturation. Forcing lipid poly-unsaturation in therapy-resistant models by combining MAPK inhibition with FASN inhibition attenuated cell proliferation and rendered cells exquisitely sensitive to a host of ROS inducers. In particular, the triple combination of MAPK inhibition, FASN inhibition, and the clinical ROS-inducing compound ATO dramatically increased survival of Mel006 PDX models from 15 to 72% with no associated signs of toxicity.

**Conclusions:**

We conclude that under MAPK inhibition the direct pharmacological inhibition of FASN evokes an exquisite vulnerability to inducers of ROS by increasing membrane lipid poly-unsaturation. The exploitation of this vulnerability by combining MAPK and/or FASN inhibitors with inducers of ROS greatly delays the onset of therapy resistance and increases survival. Our work identifies a clinically actionable combinatorial treatment for therapy-resistant cancer.

**Supplementary Information:**

The online version contains supplementary material available at 10.1186/s13046-023-02664-7.

## Background

One of the main hurdles in providing long lasting clinical benefit of targeted cancer treatments is acquired resistance to therapy. Particularly, common therapeutic approaches targeting growth factor signaling are prone to therapy escape. This is exemplified by the frequent development of resistance to MAPK inhibitors (MAPKi) in the treatment of melanoma. Multiple mechanisms of therapy resistance have been described, most converging on the re-activation of growth factor signaling pathways [1–4], often co-existing within the same lesion [5, 6]. Due to this plethora of resistance mechanisms, targeting resistant tumors is challenging. Upon combined treatment with BRAF and MEK inhibitors and despite an initial rapid and dramatic response, therapy resistance eventually develops in approximately 80% of cases [7].

In contrast to the redundancy of many signaling pathways, metabolic pathways often converge on a few key enzymes. De novo fatty acid synthesis is one emerging pathway that is increasingly shown to be critical to cancer biology [8]. Driven by key oncogenic events such as constitutive growth factor signaling and mediated by the central lipogenic transcription factor SREBP-1, the lipogenic pathway provides membrane building-blocks for cell proliferation and signaling. Importantly, it concurrently leads to the generation of saturated and (subsequently) mono-unsaturated fatty acids, leading to a general decreased poly-unsaturation of membrane lipids [9]. Since poly-unsaturated fatty acids are prone to peroxidation induced by Reactive Oxygen Species (ROS), and an accumulation of lipid hydroperoxides leads to ferroptotic cell death, the relative depletion of poly-unsaturated lipid species induced by the activation of fatty acid synthesis confers a ROS tolerant state and protects cancer cells from oxidative stress and therapeutic insults [9]. In line with this concept, we have previously shown that SREBP-1-mediated lipogenesis plays a critical role in maintaining therapy resistance in BRAF mutant melanoma and that SREBP-1 inhibition elevates lipid peroxidation, exerting a cytostatic effect [10].

Here, we sought to translate this concept into a clinically actionable therapeutic approach by using (FDA-approved and close-to-the-clinic) pharmacological agents, and to enhance the efficacy of this therapeutic strategy by supplementation with an additional ROS inducer. We provided preclinical proof-of-concept, using a Patient-Derived tumor Xenograft (PDX) model, that this novel clinically-compatible combinatorial cancer treatment can be used to overcome the development of resistance to targeted therapy in the context of BRAFV600E-melanoma, which can provide clinical relief in immunotherapy resistant patients or as salvage therapy. As the activation of fatty acid synthesis is a common occurrence in several cancer types, our findings may be generalizable to a variety of cancers such a castration resistant prostate cancer.

## Results

### Response to MAPK-inhibition correlates with lipogenic gene expression

We have previously shown that SREBP-1-mediated fatty acid biosynthesis underlies therapy resistance in BRAF-mutant melanoma [10]. However, SREBP-1 has pleiotropic functions and does not selectively control fatty acid synthesis. Instead, targeting ACACA and FASN, two downstream targets of SREBP, may more precisely control lipogenesis and represent clinically viable options.

**Fig. 1:**
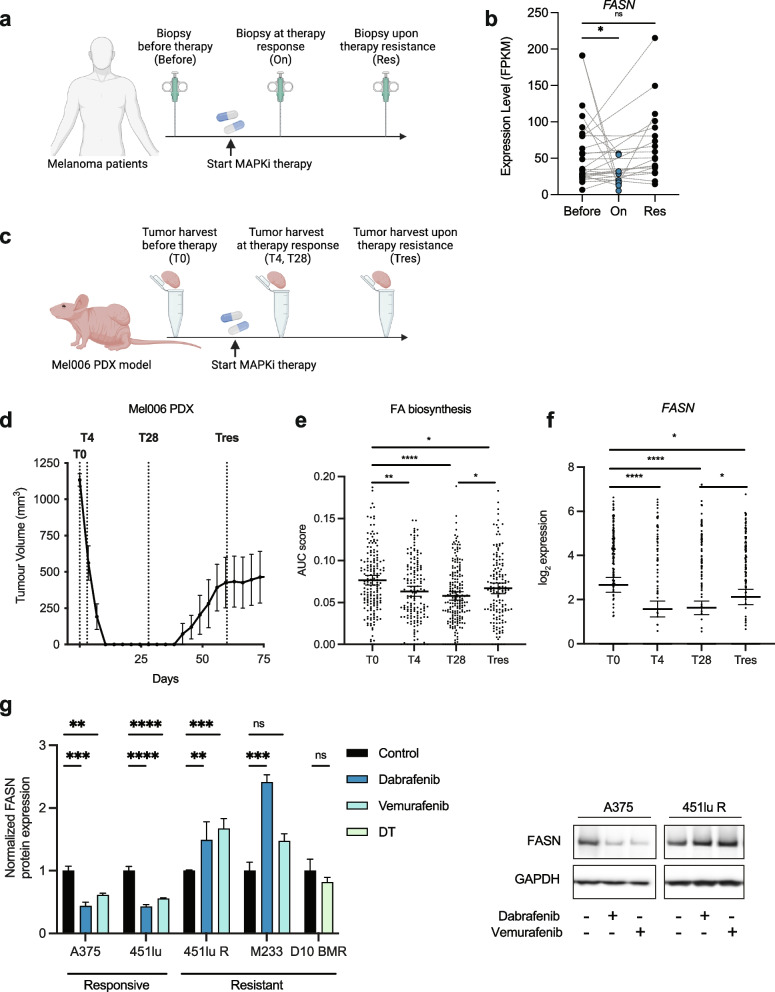
FASN expression is associated with resistance to MAPKi therapy. **a** Melanoma biopsies were taken from patients that were treatment naïve, on MAPKi treatment, and following the development of therapy resistance. **b** FASN gene expression (RNAseq) in paired samples of human melanoma before or on MAPKi therapy, and at the onset of resistance as defined by a resurgence of tumor growth. Mixed-effects analysis. **c**-**d** Mel006 PDX tumor bearing mice were treated with DT. Tumors were harvested from treatment naïve (T0, *n* = 172), on MAKPi treatment mice (T4, *n* = 155; T28, *n* = 199), and following the development of therapy resistance as defined by the resurgence of tumor growth (Tres, *n* = 148). One-way ANOVA with Tukey’s multiple comparisons. **e**–**f** Single-cell RNA-seq analysis of KEGG fatty acid biosynthesis pathway and FASN gene expression in Mel006 PDX tumors, at T0, T4, T28 and Tres. One-way ANOVA with Tukey’s multiple comparisons. **g** Relative protein expression (western blotting) of FASN in MAPKi responsive (A375, 451lu) and resistant (451lu R, M233 and D10 BMR) cells (*n* = 3) following MAPKi inhibition. One-way ANOVA with Tukey’s multiple comparisons. Right panel shows a representative western blot experiment. Data represent mean ± SEM of biologically independent samples. (*p < 0.05, **p < 0.01, ***p < 0.001, ****p < 0.0001)

In order to assess the clinical potential of this approach, we assessed the expression levels of these lipogenic targets in melanoma biopsies from treatment-naïve (Before), on MAKP inhibition treatment (On) patients, and biopsies taken following the development of therapy resistance [11] (Res). The expression of ACACA, FASN, and a host of lipogenic genes decreased upon MAPKi and recovered following the onset of resistance (Fig. 1a-b, Supplementary Figure S1a-f). Although lipid uptake is less well defined (or indeed definable) in terms of gene expression sets, the receptor CD36 is an important component. As previously described [11], the expression of CD36 followed an opposite trend and increased on treatment (Supplementary Figure S1f).

To corroborate these clinical findings, we used the BRAF-mutant PDX melanoma model Mel006, which is particularly well-suited to study mechanisms of acquired resistance to targeted therapy in melanoma [11]. Exposure to the clinically used combination of the BRAF-inhibitor dabrafenib and the MEK-inhibitor trametinib (DT) leads to rapid tumor regression, followed by a drug tolerant phase, from which resistance invariably develops (Fig. 1c, d). Interrogation of published single cell RNA-seq analysis data of Mel006 lesions harvested from drug naïve (T0), regressing (T4), drug tolerant (T28) and therapy resistant (Tres) lesions [11] showed a decline in the expression of KEGG gene-set associated with fatty acid biosynthesis at T4 and T24, followed by a recovery at Tres (Fig. 1e). This phenomenon was reflected in the expression of the lipogenic enzymes including ACACA, SCD and the flagship lipogenic gene FASN (Fig. 1f, Supplementary Figure S1g-h). Strikingly, the expression of CD36, which can play an important role in the uptake of fatty acids (including omega-3 and omega-6 poly-unsaturated fatty acids which cannot be synthesized de novo in mammalian cells) follows an opposite trend, being highly elevated at T4, and diminished at resistance (Supplementary Figure S1i). When comparing findings between clinical data and the PDX model, we conclude that the PDX model recapitulates the effect of MAPKi in lipogenesis with respect to gene expression.

We next used a panel of BRAF mutant therapy responsive and therapy resistant cell lines, namely A375 and 451 lu as therapy responsive models, and 451 lu R, M233 and D10 BMR as resistant models [12–14]. Treatment with BRAF inhibitors dabrafenib and vemurafenib reduced the expression of lipogenic enzymes including FASN in therapy responsive A375 and 451lu melanoma cell lines, but not in the isogenic therapy-resistant 451lu R, the resistant cell line M233 or the BRAF + MEK inhibitor resistant cell line D10 BMR, correlating with the effect of MAPKi on pERK and pMEK expression (Fig. 1g, Supplementary Figure S1j-l). Together, we conclude that therapy response correlates with decreased lipogenic gene expression markers.

### FASN inhibition contributes to MAPK-inhibition responsiveness in melanoma

**Fig. 2:**
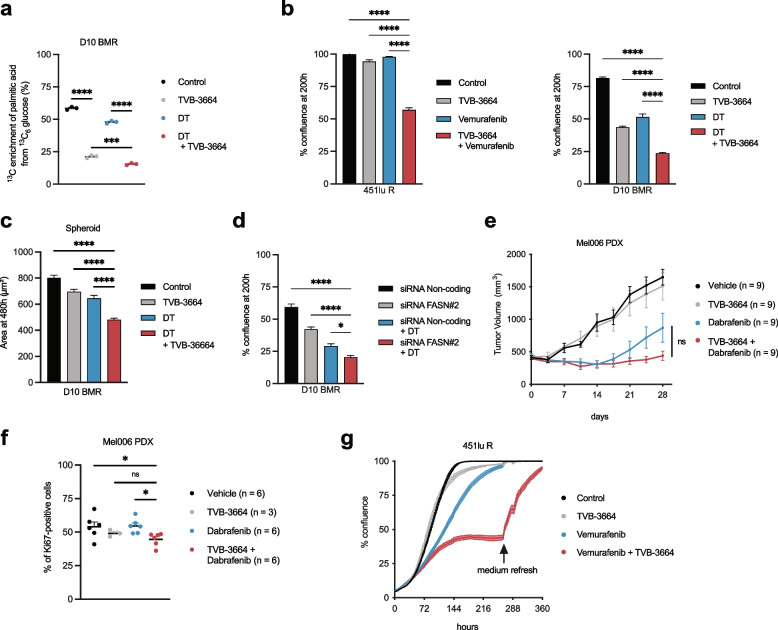
FASN inhibition sensitizes to MAPKi therapy. **a** Fraction.^13^C glucose incorporation into palmitate in D10 BMR cells (*n* = 3) following DT and TVB-3664 treatment. One-way ANOVA with Tukey’s multiple comparisons. **b**-**c** Real time cell growth analysis of 451lu R and D10 BMR cells following MAPKi and TVB-3664 treatment in 2D (*n* = 3) and 3D (*n* = 12) spheroid cultures. One-way ANOVA with Tukey’s multiple comparisons. **d** Real time cell growth analysis of D10 BMR cells (*n* = 6) following DT treatment and siRNA-mediated knockdown of FASN. One-way ANOVA with Tukey’s multiple comparisons. **e** Tumor growth curves of Mel006 PDX bearing mice treated with vehicle, TVB-3664, dabrafenib, or the combination. One-way ANOVA with Tukey’s multiple comparisons. **f** Ki67 staining of Mel006 PDX bearing mice treated with vehicle, TVB-3664, dabrafenib, or the combination. One-way ANOVA with Tukey’s multiple comparisons. **g** Real time cell growth analysis of 451lu R cells (*n* = 4) following MAPKi and TVB-3664 treatment with medium refresh (without treatment) at indicated timepoint. Data represent mean ± SEM of biologically independent samples. (*p < 0.05, **p < 0.01, ***p < 0.001, ****p < 0.0001)

Reasoning that fatty acid synthesis and the subsequent relative reduction of membrane lipid poly-unsaturation are important for the development of therapy resistance, we inhibited this pathway in DT-resistant 451lu R and D10 BMR melanoma cells in combination with the potent and selective FASN inhibitor TVB-3664 [15]. To demonstrate the activity of TVB-3664, we assessed its effect on lipogenesis in therapy-resistant D10 BMR cells using ^13^C_6_-glucose tracing into fatty acids. TVB-3664 reduced de novo palmitate synthesis by 3-fold and MAPK targeting in combination with TVB-3664 slightly further diminished palmitate synthesis (Fig. 2a). Similar effects were observed in 451lu R cells using ^13^C_6_-glucose tracing into lipids under MAPK and FASN inhibition (Supplementary Figure S2a). Consistent with the involvement of de novo fatty acid synthesis in the production of saturated and mono-unsaturated fatty acids [9], FASN inhibition increased the propensity of D10 BMR cells to take up exogenous fatty acids as measured by media palmitate depletion (Supplementary Figure S2b).

Using live cell imaging, we showed that exposure of 451lu R and D10 BMR cells to the combination of MAPKi and TVB-3664 diminished cell proliferation when cells were grown in either 2D plasticware or as spheroids (Fig. 2b, c). Although these cells are resistant to therapy, MAPKi slows the growth of the cells, but to a dramatically lesser extent when compared to therapy-responsive cells. In order to show that these effects are not cell line dependent and/or mediated by off-target effects, we firstly confirmed findings using the additional cell line M233, and used the ACACA inhibitor soraphen A in both 451lu R and D10 BMR cells (Supplementary Figure S3). Moreover, knockdown of FASN and ACACA using multiple siRNA constructs was consistent with these findings (Fig. 2d, Supplementary Figure S4). Although MAPKi is generally an effective strategy in BRAF mutant melanoma, it is of no clinical benefit in a BRAF WT background. To this end, using the BRAF WT cells M202, M207 and M257, we next showed that MAPKi and FASN inhibition cooperate in reducing cell growth and suggests some utility in using this combination treatment (Supplementary Figure S5).

In order to assess the effect of FASN inhibition on MAPK inhibitor response in a more translational model, we treated Mel006 PDX with TVB-3664 in a setting of BRAF-targeting monotherapy which in contrast to DT (Fig. 2e) exerts a transient inhibitory effect on Mel006 tumors, allowing a short-term study set-up. Whereas tumor volume measurements in Mel006 bearing mice showed that dabrafenib treatment combined with TVB-3664 resulted in a non-significant volume decrease during the course of the study, Ki67 staining of Mel006 tumors revealed that BRAF inhibition combined with FASN inhibition reduces tumor cell proliferation (Fig. 2f). We conclude that the combination of MAPK and FASN inhibition is not sufficient to drive a potent antitumor effect. These findings are supported by the cell line growth kinetics in 451luR cells. Importantly, refreshing media at late time points rescues cell proliferation, indicating that the effects are cytostatic, rather than cytotoxic (Fig. 2g).

Together, we conclude that lipogenesis contributes to therapy response/resistance in melanoma.

### MAPK-inhibition and FASN inhibition promote membrane lipid poly-unsaturation

**Fig. 3:**
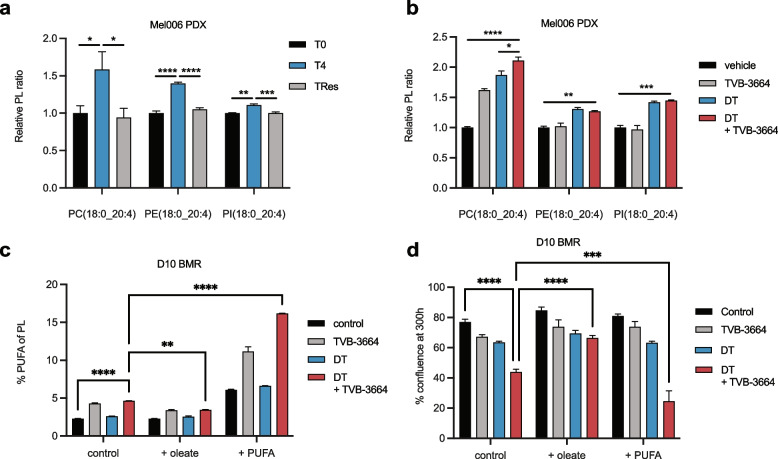
FASN inhibition drives membrane lipid poly-unsaturation. **a** Relative abundance of indicated lipid species in Mel006 tumors harvested at T0 (*n* = 6), T4 (*n* = 4) and Tres (*n* = 5). One-way ANOVA with Tukey’s multiple comparisons. **b** Relative abundance of indicated lipid species in Mel006 tumors following MAPKi and TVB-3664 treatment (*n* = 3). One-way ANOVA with Tukey’s multiple comparisons. **c** Phospholipidomics of PC, PE, PI, PS and PG species in D10 BMR cells (*n* = 3) treated with DT, TVB-3664 or DT + TVB-3664 in combination with exposure with oleate, palmitate or a 1:1 mixture of PUFA (linoleate and linolenate). Two-way ANOVA with Tukey’s multiple comparisons. **d** Real time cell growth analysis of D10 BMR cells (*n* = 3) with supplementation of exogenous palmitate, oleate, or PUFA at 300 h. Two-way ANOVA with Tukey’s multiple comparisons. Data represent mean ± SEM of biologically independent samples

Consistent with the downregulation of lipogenic gene expression upon therapy exposure and a concomitant increase in fatty acid uptake, we observed a transient increase at T4 in the amount of poly-unsaturated membrane phospholipids species as exemplified by eicosatetraenoic acid (20:4, a fatty acid that cannot be synthesized de novo in mammalian cells) in Mel006 tumors (Fig. 3a, Supplementary Figure S6). Importantly, the extent of membrane lipid poly-unsaturation could be modulated by treating Mel006 bearing mice with MAPKi, the FASN inhibitor TVB-3664 and especially when used in combination (Fig. 3b, Supplementary Figure S7).

When using cell lines, likely due to less heterogeneity and the lack of a stromal component, membrane lipid changes were even more pronounced. Specifically, FASN inhibition increased relative membrane lipid poly-unsaturation in D10 BMR cells, which was further elevated by co-treatment with MAPK inhibitors (Fig. 3c). The resulting phenotypic effects on membrane fluidity/disorder were measured by Di-4-ANEPPDHQ and are consistent with the lipidomics data in that FASN inhibition augments membrane disorder and cooperates with MAPK inhibition to further increase membrane disorder in 451lu R and in D10 BMR cells (Supplementary figure S8). Importantly, the poly-unsaturation effect of combined MAPK and FASN inhibition could be modulated by the addition of exogenous oleate or a mixture of linoleate and linolenate. We next recapitulated these findings in 451lu and in 451lu R cells and thereby show that they are not cell-line dependent (Supplementary Figure S9). Importantly, under MAPKi, exposure to oleate (a major end-product of fatty acid biosynthesis) reversed some of the cell growth inhibitory effects, in D10 BMR cells, whereas exposure to a mixture of linoleate and linolenate did not (Fig. 3d, Supplementary Figure S10). We conclude that MAPKi cooperates with FASN inhibition in driving a switch from lipogenesis towards exogenous lipid uptake, and in doing so, promotes membrane lipid poly-unsaturation by virtue of their relative exogenous abundance, which contributes to therapy response. Taken together, targeted therapy in melanoma causes a reduction of lipogenic gene expression and a concomitant increase in membrane lipid poly-unsaturation, with consequences for cell proliferation, which can be further modulated by the addition of exogenous lipids. We therefore conclude that the degree of membrane lipid poly-unsaturation contributes to therapy response in melanoma models.

### Inhibition of fatty acid synthesis sensitizes therapy-resistant melanoma to ROS exposure

**Fig. 4:**
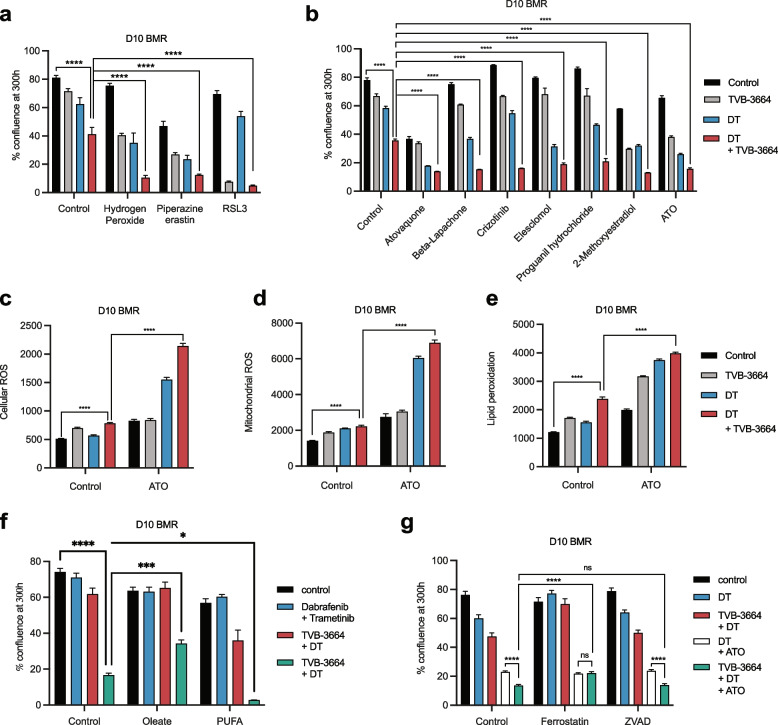
FASN inhibition increases sensitivity of melanoma cell lines to clinical ROS inducers. **a** Real time cell growth analysis of D10 BMR cells (*n* = 3) treated with DT, TVB-3664 and hydrogen peroxide, piperazine erastin or RSL3. Two-way ANOVA with Tukey’s multiple comparisons. **b** Real time cell growth analysis of D10 BMR cells treated with DT, TVB-3664 in combination with 2-methoxyestradiol (*n* = 2), atovaquone (*n* = 2), beta-lapachone (*n* = 2), crizotinib (*n* = 2), elesclomol (*n* = 2), proguanil hydrochloride (*n* = 3) or ATO (*n* = 3). Two-way ANOVA with Tukey’s multiple comparisons. **c**-**e** Cellular ROS**,** mitochondrial superoxide and oxidized C11 BODIPY levels as a surrogate of lipid peroxidation degree in D10 BMR cells (*n* = 6) treated with DT, ATO and TVB-3664 at 120 h. **f**-**g** Real time cell growth analysis of D10 BMR cells treated with DT, TVB-3664 and ATO, in combination with oleate (*n* = 12), PUFA (*n* = 9), ferrostatin-1 (*n* = 15) or ZVAD (*n* = 15). Two-way ANOVA with Tukey’s multiple comparisons. Data represent mean ± SEM of biologically independent samples. (*p < 0.05, ***p < 0.001, ****p < 0.0001)

Polyunsaturated fatty acids (PUFA) are prone to lipid peroxidation. Specifically, the peroxidation of PUFA-phospholipids is linked to cell damage and to increased ROS and to ferroptosis [9, 16, 17]. Considering that exogenous lipids contain more PUFA species that endogenously synthesized lipids which are mostly saturated and mono-unsaturated, we reasoned the lipogenesis inhibition would drive compensatory lipid uptake. In this way, we hypothesized that FASN-inhibition may render melanoma cells more sensitive to ROS by increasing the relative amount of membrane PUFA-phospholipids. Consistent with our hypothesis, treating 451lu R and D10 BMR cells with DT and TVB-3664 sensitized them to treatment with hydrogen peroxide (Fig. 4a, Supplementary Figure S11). Similarly, as both SLC7A11 and GPX4 have been shown to serve critical antioxidant roles especially in response to phospholipid hydroperoxides, accordingly, MAPKi and TVB-3664 treatment rendered D10 BMR and 451lu R sensitive to chemical modulators of these targets (piperazine erastin and RSL3) (Fig. 4a, Supplementary Figure S11). Moreover, exposure of D10 BMR cells with DT and TVB-3664 sensitized them to treatment with a host of FDA approved or in clinical trials ROS elevating drugs, specifically, 2-methoxyestradion, atovaquone, beta-lapachone, crizotinib, elesclomol, proguanil hydrochloride and ATO [18, 19] (Fig. 4b, Supplementary Figure S12). Taken together, fatty acid synthesis and MAPKi render melanoma cells particularly sensitive to ROS elevating drugs.

Considering the FDA approval status of ATO, its potent observed in vitro effect and that it is well tolerated clinically [19], we selected this drug for further study. ATO potently sensitized the growth inhibitory effects MAPKi and TVB-3664 treatment in 451lu R and in D10 BMR cells (Supplementary Figure S12g-h).

ROS measurement in D10 BMR cells revealed that MAPKi and ATO elevated both cellular and mitochondrial ROS and the amount of oxidized BODIPY-C11 (as surrogate of lipid peroxidation), and further synergized with FASN inhibition. The elevation was most pronounced in the triple combination treatment (Fig. 4c-e). There were no significant effects of this treatment combination on mitochondrial membrane potential (Supplementary Figure S13) Modulating the membrane lipid profile of D10 BMR by the addition of exogenous oleate or PUFA in D10 BMR cells affected their response to ATO, suggesting that membrane lipid polyunsaturation drives sensitivity to ATO treatment and thereby to ROS elevation (Fig. 4f, Supplementary Figure S14). Likewise, treating D10 BMR cells with the lipid soluble antioxidant ferrostatin-1 but not ZVAD fully rescued the effects of DT + TVB-3664 + ATO versus DT + ATO combination treatment (Fig. 4g, Supplementary File S15). Taken together, our data suggests that the functional deleterious effects of combined DT + TVB-3664 + ATO treatment is in part driven by membrane lipid peroxidation.

### A salvage combination treatment to overcome resistance to targeted therapy in melanoma

**Fig. 5:**
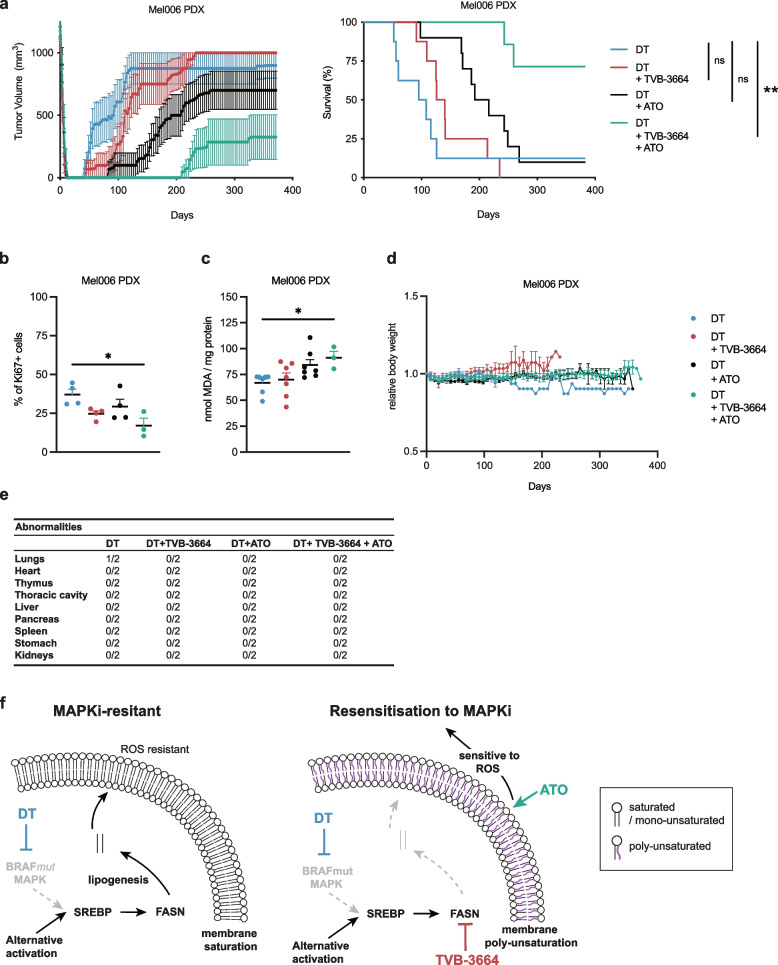
A clinically relevant combination therapy delays onset of acquired resistance. **a** Tumor growth curve and sacrifice time-point (percent survival) in Mel006 PDX tumor bearing mice following treatment with DT (*n* = 8), DT + TVB-3664 (*n* = 8), DT + ATO (*n* = 10) or DT + TVB-3664 + ATO (*n* = 7). Log-rank (Mantel-Cox) test. **b** % of Ki67 cancer cells in harvested tumors (*n* = 4, except for the DT + TVB-3664 + ATO condition where only 3 were available). One-way ANOVA with Tukey’s multiple comparisons. **c** MDA abundance in harvested tumors (*n* = 7, except for the DT + TVB-3664 + ATO condition where only 3 were available). One-way ANOVA with Tukey’s multiple comparisons. **d** Mouse body weight changes in Mel006 PDX tumor bearing mice following treatment with DT (*n* = 8), DT + TVB-3664 (*n* = 8), DT + ATO (*n* = 10) or DT + TVB-3664 + ATO (*n* = 7). **e** Summary of observed abnormalities in Mel006 tumor bearing mice. **f** Sustained MAPK signaling in therapy resistant melanoma cells sustains lipogenesis and generates saturated and monounsaturated fatty acid species which are incorporated into membranes, thereby saturating membranes. This phenomenon renders the cancer cell resistant to ROS mediated membrane lipid peroxidation. Pharmacological inhibition of FASN reverses this effect by blocking lipogenesis and thereby increasing lipid uptake. As exogenous lipids are more enriched in polyunsaturated species, this effect poly-unsaturated membranes, renders the cancer cells ROS sensitive, and can form an actionable clinical strategy. Data represent mean ± SEM. (*p < 0.05, **p < 0.01)

We exposed treatment-naive Mel006 melanoma bearing mice to DT, TVB-3664, ATO and TVB-3664 + ATO once tumors reached a volume of 1000 mm^3^. A pilot study using treatments without the combination with DT did not show any tumor growth delay and hence these conditions were no longer included in the final experiment to reduce the number of animals (Supplementary Figure S16). Upon treatment with DT, tumors initially shrunk to undetectable volumes and reappeared as early as 40 days after start of treatment (Fig. 5a). The combination of TVB-3664 with DT delayed tumor reappearance in a fraction of the animals, although the tumors caught up at later time points. Combination of DT with ATO had a more pronounced and more lasting effect with an average delay in growth of 40 days. The most striking effect was observed in the triple combination where five/seven mice were entirely tumor free, and after a follow up period of 375 days, tumor regrowth was observed in a total of three/seven mice (Fig. 5a, Supplementary Figure S16). Moreover, by continuing therapy even in the event of resistance, tumors grew more slowly, as further evidenced by tumor growth curves and decreased Ki67-staining in those tumors (Fig. 5a-b). In this condition, after 375 days, a survival of 72% of the animals was reached in the combination cohort, compared to a mere 15% in all other conditions. The amount of the lipid hydroperoxide degradation product MDA significantly increased in Mel006 tumors in the triple combination treatment over DT and DT + TVB3664, and non-significantly increased over DT + ATO (Fig. 5c). Mouse body weight did not significantly differ across cohorts (Fig. 5d). Moreover, comprehensive pathological analysis of the cohort revealed only mild pathologies in some mice including mild portal hepatitis and viral bronchitis, but these did not correlate with any specific treatment combination and are expected in ageing mice, suggesting that the combination therapy is well-tolerated (Fig. 5e, and Supplementary File 1). Taken together, these data identify a novel, clinically actionable and well-tolerated combination treatment that effectively delays the occurrence of resistance and dramatically increases progression-free survival in BRAF mutant melanoma (Fig. 5f).

## Discussion

Although targeted therapies typically offer significant therapeutic relief, myriad resistance mechanisms render this short lived. In the context of BRAF mutant melanoma, here, we demonstrate the applicability of a lipid metabolic additive therapy combination that exploits acquired vulnerabilities in augmenting a therapy response. Using clinically actionable compounds, we demonstrate that inhibition of lipogenesis in combination with MAPK therapy renders cancer cells exquisitely sensitive to ROS inducers. In a PDX model of therapy resistance, we show an increase of survival from 40 to 375 days, wherein there is no tumour resurgence throughout the experiment in most combination therapy mice.

Besides its commonly reported role in membrane biogenesis in rapidly proliferating cancer cells [20], accumulating evidence supports the crucial involvement of fatty acid synthesis in the protection of cancer cells from oxidative and therapeutic stress [9, 21]. This protective effect is related to the synthesis of saturated and mono-unsaturated fatty acids and the subsequent relative decrease in poly-unsaturated lipids which are prone to lipid peroxidation and play a central role in ferroptosis [9, 17]. Moreover, both therapy resistant persister cells and mesenchymal cancer cells which are notoriously resistant to therapies appear to be responsive to ferroptosis [22, 23]. In the context of melanoma, we and others have previously reported that sustained SREBP-1-dependent lipogenesis maintains therapy resistance in melanoma [10, 24]. However, no clinical-grade pharmacological inhibitors of SREBP-1 are currently available. Moreover, inhibition of lipogenesis per se may be insufficient to eradicate cancer cells as demonstrated in the current study.

In order to exploit the translational potential of these findings, we developed a clinically actionable strategy by selecting a combination strategy using FDA-approved and in clinical trial pharmacological agents. Although pre-clinical inhibitors to several lipogenic enzymes such as ACACA and SCD are in pre-clinical use, none are closer to the clinic than those targeting FASN. Specifically, we selected the FASN inhibitor TVB-3664 which is the mouse compatible analogue of TVB-2640, a first-in-class orally bioavailable FASN inhibitor in Phase 2 clinical trial for nonalcoholic steatohepatitis (NASH) and with potential antineoplastic indications [25, 26]. Inhibiting FASN forces cancer cells to switch towards lipid uptake, which are enriched in ROS labile PUFA, which we exploit by the addition of a ROS elevating drug.

Mechanistically, we propose that lipid peroxidation and ferroptotic cell death can in part explain this phenomenon, as evidenced by the in vitro rescue effects using ferrostatin-1 but not ZVAD. Consistent with our finding, multiple studies show that membrane enrichment in PUFA species is a potent predictor of ferroptosis sensitivity [17, 27–30]. Importantly, although many treatment-resistant cancers have acquired mechanisms to evade apoptosis, they typically show sensitivity to ferroptosis inducers [22, 23]. As prototypical ferroptosis inducers such as erastin and RSL3 to date have limited clinical potential, we have selected arsenic trioxide (Trisenox/ATO) as a ROS inducer as it is in clinical use for acute promyelocytic leukaemia (APL) with acceptable toxicity (as also alluded to also in this study). Future work may focus on the exploration of other ROS inducers, including iron supplementation, thereby exploiting dysregulated iron metabolism as another exploitable vulnerability in many cancers [31, 32]. It will also be interesting to explore the benefit of dietary fatty acid modulation to work in concert with this approach. This is particularly tantalizing in highly hypoxic cancers, as under oxygen deficient conditions, MUFA generation cannot sufficiently occur, and in order to maintain membrane homeoviscosity, cells may require compensation by exogenous PUFA uptake [33]. Future challenges lie in the potential acquisition of mechanisms by which cancer cells may reduce the expose of PUFA to ROS-induced lipid peroxidation. One mechanism involves the activation of phospholipases that preferentially cleave membrane PUFA and thereby render cells more resistant to ferroptosis [34, 35]. Also, the spatial distribution of PUFA in the cell may control ferroptosis sensitivity. Specifically, sequestering membrane-PUFA into triglycerides pools renders them less accessible to ROS and thereby render cells resistant to ferroptosis [36, 37]. Of interest, near-clinical grade inhibitors of these pathway are available to further sensitize cells to ROS-induced lipid peroxidation [38, 39].

## Conclusion

Taking BRAF-mutant melanoma as paradigm, we demonstrate that FASN expression is consistently increased upon onset of therapy resistance and is associated with decreased lipid poly-unsaturation. Under MAPK inhibitor treatment, pharmacological inhibition of FASN evokes an exquisite vulnerability to inducers of ROS by increasing membrane lipid poly-unsaturation. Exploitation of this vulnerability by combining MAPK and FASN inhibitors with inducers of ROS greatly delays the onset of therapy resistance and increases survival (Fig. 5f). Considering that besides the MAPK pathway, multiple oncogenic drivers such as EGFR [40, 41] or the androgen receptor [42, 43] promote fatty acid synthesis, the here proposed combinatorial treatment may be applicable to several tumor types. As a number of ROS-elevating drugs are FDA approved and FASN inhibitors are in clinical development, these findings have potential for a swift translation to a clinical setting.

## Methods

### Cell culture

A375 were obtained from ATCC. M202, M207, M257 and M233 were gifted by Professor A. Ribas. 451 and 451lu R, were gifted by Professor R. Lo. D10 BMR were gifted by Professor Daniel Peeper. All cell lines were propagated in DMEM High Glucose (Sigma), supplemented with 10% FBS (Gibco) and 4 mM glutamine (Thermo Fisher Scientific). 451lu R growth media was supplemented with vemurafenib (0.5 μM). D10 BMR growth media was supplemented with dabrafenib (2.5 μM and trametinib (0.5 μM). All cell cultures were periodically tested for mycoplasma contamination. All experiments were performed in DMEM High Glucose, supplemented with 2% FBS (Thermo Fisher Scientific) and 4 mM glutamine, except for ^13^C_6_-glucose metabolite tracer studies, where 4.5 g L − 1 ^13^C-glucose (Cambridge isotope laboratories) was supplemented to DMEM no glucose (Thermo Fisher Scientific). The following compounds were used at the stated concentrations unless otherwise indicated. Dabrafenib (5 μM in M202, M207, M257, 451lu R and M233 cells, 2.5 μM in D10 BMR cells) from Adooq Bioscience, trametinib (0.5 μM) from MedChem Express, vemurafenib (5 μM) from ApexBio, TVB-3664 (40 nM) from Sagimet Biosciences (formerly 3 V Biosciences), soraphen A (50 nM) (provided by Drs. Klaus Gerth and Rolf Jansen (Helmholtz-Zentrum für Infektionsforschung, Braunschweig, Germany)), piperazine erastin from MedChem Express (7.5 μM). RSL3 (5 μM), arsenic trioxide (4 μM), hydrogen peroxide (250 μM for D10 BMR and 600 μM for 451lu R), oleate (40 μM), linoleate (20 μM), linolenate (20 μM), 2-Methoxyestradiol (1 μM), atovaquone (10 μM), beta-lapachone (400 nM), crizotinib (630 nM), elesclomol (200 nM), proguanil hydrochloride (10 μM), ferrostatin-1 (5 μM), NAC (120 μM) and ZVAD (10 μM) were all obtained from Sigma.

### RNA-seq

Single cell RNAseq data of PDX model derived melanoma cells was interrogated for FASN expression during different MAPKi exposure times (GEO: GSE116237) [11].

### Gene knockdown studies

D10 BMR cells were reverse transfected with 100 nM of Silencer-Select Negative Control siRNA (Thermo Fischer Scientific), three sequences targeting ACACA and three targeting FASN (Silencer Select siRNA, Thermo Fischer Scientific) with lipofection according to the manufacturer’s recommendations (Lipofectamine RNAiMAX, Thermo Fischer Scientific).

### RNA extraction and qRT-PCR

RNA extraction and qRT-PCR were performed as described previously [44]. Primers used were: FASN: Fw 5′-TCCGAGATTCCATCCTACGC-3′, Rv 5′-GCAGCTGTGACACCTTCAGG-3′, ACACA: Fw 5′-TGAACTTCACACAGGTAGTCTGCC-3′, Rv 5′-TGGAACACTCGATGGAGTTTCT-3′, and 18 S as a reference gene: Fw 5′-CGCCGCTAGAGGTGAAATTC-3′, Rv 5′-TTGGCAAATGCTTTCGCTC -3′.

### Fatty acid uptake

#### Metabolite extraction and derivatization method

Metabolites were extracted from the cell culture media at the beginning (t0) and at the end (t1) of the experiment as described before [45, 46]. Briefly, 200 µl of media samples were resuspended with 800 µl of 62.5% methanol containing 90 ng/ml of glutaric acid precooled in a mixture of dry and wet ice. Subsequently, 500 µL of precooled chloroform containing 10 µg/ml of C17 internal standard were added and samples were vortexed for 10 min at 4 °C followed by a centrifugation for other 10 min (max. speed, 4 °C). After centrifugation, polar metabolites in the methanol/water (upper) phase and the lipid fraction in the chloroform (lower) phase were separated. The lipid fraction was dried at 4 °C overnight using a vacuum concentration.

The samples were derivatized and measured as described before [47–49]. Briefly, fatty acids were esterified with 500 µL of 2% sulfuric acid in methanol buffer per sample and incubated overnight at 50 °C. Subsequently, fatty acids were extracted with 600 µl of MS-grade hexane and 100 µl of saturated NaCl. Hexane fraction was dried in a vacuum concentration at room temperature for 30 min and was resuspended in 50 µl of hexane.

#### Gas chromatography–mass spectrometric analysis

The metabolites were analyzed by gas chromatography (8860 GC system) coupled to mass spectrometry (5977B Inert MS system) from Agilent Technologies. The inlet temperature was set at 270 °C and 1 µL of samples were injected in splitless mode. Metabolites were separated with a DB-FASTFAME column (30 m × 0.250 mm). Helium was used as a carrier gas with a flow rate of 1 mL/min. For the separation of fatty acids, the initial gradient temperature was set at 50 °C for 1 min and increased at the ramping rate of 12 °C/min to 180 °C, following by a ramping rate of 1 °C/min to rich 200 °C. Finally, the final gradient temperature was set at 230 °C with a ramping rate of 5 °C/min for 2 min. The temperatures of the quadrupole and the source were set at 150 °C and 230 °C, respectively. An electron impact ionization fixed at 70 eV was applied and a full scan mode was used for the measurement of fatty acid, ranging from 100 to 400 a.m.u (mass).

#### Data analysis – Matlab

After the acquisition of raw ion chromatograms through MSD Chemstation Data Analysis, a Matlab M-file was used to extract mass distribution vectors, then the different metabolites were integrated and the peak area was subsequently normalized to the volume of media extracted and to the internal standard C17. In order to calculate uptake/secretion, we subtracted the metabolite abundances extracted from the media at t0 with the metabolites abundances extracted from the media at t1; the data were further normalized to the DNA content of the cultured cells present at t1.

### Lipidomics

#### Lipid extraction

700 μl of sample (4 μl of plasma diluted in water, or 700 μl of homogenized cells) was mixed with 800 μl 1 N HCl:CH3OH 1:8 (v/v), 900 μl CHCl3, 200 μg/ml of the antioxidant 2,6-di-tert-butyl-4-methylphenol (BHT; Sigma Aldrich) and 3 μl of SPLASH® LIPIDOMIX® Mass Spec Standard (Avanti Polar Lipids, #330,707). After vortexing and centrifugation, the lower organic fraction was collected and evaporated using a Savant Speedvac spd111v (Thermo Fisher Scientific) at room temperature and the remaining lipid pellet was stored at—20 °C under argon.

#### Mass spectrometry

Just before mass spectrometry analysis, lipid pellets were reconstituted in 100% ethanol. Lipid species were analyzed by liquid chromatography electrospray ionization tandem mass spectrometry (LC-ESI/MS/MS) on a Nexera X2 UHPLC system (Shimadzu) coupled with hybrid triple quadrupole/linear ion trap mass spectrometer (6500 + QTRAP system; AB SCIEX). Chromatographic separation was performed on a XBridge amide column (150 mm × 4.6 mm, 3.5 μm; Waters) maintained at 35 °C using mobile phase A [1 mM ammonium acetate in water-acetonitrile 5:95 (v/v)] and mobile phase B [1 mM ammonium acetate in water-acetonitrile 50:50 (v/v)] in the following gradient: (0–6 min: 0% B → 6% B; 6–10 min: 6% B → 25% B; 10–11 min: 25% B → 98% B; 11–13 min: 98% B → 100% B; 13–19 min: 100% B; 19–24 min: 0% B) at a flow rate of 0.7 mL/min which was increased to 1.5 mL/min from 13 min onwards. SM, CE, CER, DCER, HCER, LCER were measured in positive ion mode with a precursor scan of 184.1, 369.4, 264.4, 266.4, 264.4 and 264.4 respectively. TAG, DAG and MAG were measured in positive ion mode with a neutral loss scan for one of the fatty acyl moieties. PC, LPC, PE, LPE, PG, PI and PS were measured in negative ion mode by fatty acyl fragment ions. Lipid quantification was performed by scheduled multiple reactions monitoring (MRM), the transitions being based on the neutral losses or the typical product ions as described above. The instrument parameters were as follows: Curtain Gas = 35 psi; Collision Gas = 8 a.u. (medium); IonSpray Voltage = 5500 V and − 4,500 V; Temperature = 550 °C; Ion Source Gas 1 = 50 psi; Ion Source Gas 2 = 60 psi; Declustering Potential = 60 V and − 80 V; Entrance Potential = 10 V and − 10 V; Collision Cell Exit Potential = 15 V and − 15 V.

The following fatty acyl moieties were taken into account for the lipidomic analysis: 14:0, 14:1, 16:0, 16:1, 16:2, 18:0, 18:1, 18:2, 18:3, 20:0, 20:1, 20:2, 20:3, 20:4, 20:5, 22:0, 22:1, 22:2, 22:4, 22:5 and 22:6 except for TGs which considered: 16:0, 16:1, 18:0, 18:1, 18:2, 18:3, 20:3, 20:4, 20:5, 22:2, 22:3, 22:4, 22:5, 22:6.

#### Data analysis

Peak integration was performed with the MultiQuant™ software version 3.0.3. Lipid species signals were corrected for isotopic contributions (calculated with Python Molmass 2019.1.1) and were quantified based on internal standard signals and adheres to the guidelines of the Lipidomics Standards Initiative (LSI) (level 2 type quantification as defined by the LSI).

### Immunoblotting analysis

Following ice-cold PBS washes, cells were collected in sample buffer (Thermo Fisher Scientific) supplemented with DTT (Sigma), sonicated and boiled for 5 min. Equal amounts of protein were loaded onto precast gels (NuPAGE, Thermo Fisher Scientific), transferred to nitrocellulose membranes, and incubated with antibodies against ACACA (1/1000 dilution) (Cell Signaling, # 3676), FASN (1/1000 dilution) (Cell Signaling, #3180), phospho-MEK1/2 ser217/221 (1/1000 dilution) (Cell Signaling, #9154), phospho-ERK1/2 Thr202/Tyr204 (1/1000 dilution) (Cell Signaling, # 9101) and GAPDH (1/20000 dilution) (Cell Signaling, #5174). Full unedited blots are shown in Supplementary Data File 1.

### Histological staining and analysis

The following antibody was used for detecting the following protein: anti-Ki67 (rabbit, 1:1000, Thermo Fischer Scientific, #RM-9106-S). Furthermore, the PerkinElmer Opal 4-Color Manual IHC Kit (PerkinElmer/Akoya, NEL810001KT) was used for the tyramide signal amplification according to the manufacturer’s protocol. For introduction of the secondary-HRP the Envision + /HRP goat anti-Rabbit (Dako Envision + Single Reagents, HRP, Rabbit, Code K4003) was used for antibody raised in rabbit (Ki67). The protein Ki67 was detected using the OPAL 570 reagent. Images were acquired on the Zeiss Axio Scan.Z1 using a × 20 objective and ZEN 2 software. For exporting images the ZEN 2 software (Zeiss) was used.

### Proliferation assays

Proliferation curves were generated using an IncuCyte ZOOM system (Essen BioScience) on cells seeded on microplates (TPP or Nunc Edge) based on phase contrast images taken at 2 h intervals for the duration of the experiments, except for Supplementary figure S16 where plates were scanned once per day. Spheroid growth assays were performed in Nunclon™ Sphera™ Microplates (Thermo Fisher Scientific). Cells were seeded at a density of 8000 cells per well (except for M202, M207 and M257 cells which were seeded at 32,000 cells per well) in 96 well plates or 150 cells per spheroid.

### Flow cytometric analysis

Cellular, Mitochondrial ROS and mitochondrial membrane potential were measured using the CellROX Deep red, MitoSOX red mitochondrial superoxide indicator and JC-1 respectively (Thermo Fisher Scientific) according to the manufacturer’s instructions, except half the recommended dye concentration was used. Unused dye was flushed under Argon or nitrogen and stored for re-use. Membrane order was measured using Di-4-ANEPPDHQ (Thermo Fisher Scientific) at a concentration for 1 µg per ml and incubated for half an hour. Lipid peroxidation potential was measured using BODIPY™ 581/591 C11 (Thermo Fisher Scientific) according to the manufacturer’s instructions. Cells were assayed using a FACS Verse flow cytometer (BD Biosciences).

### MDA measurements

A TBA solution was prepared using 5.2 mg of 2-Thiobarbituric acid (Sigma), 50 mg of butylhydroxytoluene as antioxidant (Sigma) in 30% (v/v) glacial acetic acid (Sigma) in water (Baxter) to 50 ml. Solution was sonicated and pH was adjusted by dropwise sodium hydroxide solution (Sigma) addition to 3.5 in order to render it safer to handle. MDA standard (Sigma) and tumor homogenates were heated (95 °C) in the TBA solution for an hour before absorbance was measured using a plate reader (Perkin Elmer). Results were normalized to total cellular DNA.

### Animal experiments

The cutaneous melanoma PDX models are part of the Trace collection (https://www.uzleuven-kuleuven.be/lki/trace/trace-leuven-pdx-platform) and were established using metastatic melanoma lesions derived from patients undergoing surgery as part of the standard treatment at the UZ Leuven. Written informed consent was obtained from patients and all procedures involving human samples were approved by the UZ Leuven/KU Leuven Medical Ethical Committee (S54185) and carried out in accordance with the principles of the Declaration of Helsinki. The experiments were approved by the KU Leuven animal ethical committee under ECD P038-2015 and performed in accordance with the internal, national and European guidelines of Animal Care and Use. Tumors fragments derived from the established Mel006 PDX model (generation F ≥ 3) were implanted subcutaneously in the interscapular fat pad of female mice (NMRI-Fox1nu, Taconic).

In the experiment in Fig. 2E: When tumor size reached 500 mm^3^, mice were randomly assigned to a cohort and drugs or vehicles were blindly administered daily by oral gavage. Dabrafenib (30 mg / kg) was administered by daily oral gavage in 10% DMSO (Sigma) in 90% PBS (Thermo Fischer Scientific). TVB-3664 (1 mg / kg) was administered by daily oral gavage as an emulsion of 30% Polyethylene glycol 400 (Sigma), in water (Baxter). Tumor size was measured blindly with digital calipers (Fowler Sylvac) twice a week. The investigators were blinded for the evaluation of the results.

In the experiment in Fig. 5A: When tumor size reached 1000 mm^3^, mice were randomly assigned to a cohort and drugs or vehicles were blindly administered daily by oral gavage. Dabrafenib (30 mg / kg) and trametinib (0.3 mg / kg) were administered in a single solution by daily oral gavage in 10% DMSO (Sigma) in 90% PBS (Thermo Fischer Scientific). TVB-3664 (1 mg / kg) was administered by daily oral gavage as an emulsion of 30% Polyethylene glycol 400 (Sigma), in water (Baxter). Arsenic trioxide was administered by IP injection three times a week (6 mg / kg). Tumor size was measured blindly with digital calipers (Fowler Sylvac) twice a week. The investigators were blinded for the evaluation of the results.

### Statistical analysis

The results were analyzed in GraphPad Prism 6.0 h using a t-test. In case of multiple comparisons, a correction was applied using the Holm–Sidak method. *p*-values of < 0.05 were considered to be statistically significant (*p < 0.05, **p < 0.01, ***p < 0.001, ****p < 0.0001).

For non-gaussian data the Kruskal–Wallis test was used. All data presented represent means ± SEM unless stated otherwise in the figure legend. Figures presented are representative experiments of based on biological triplicates, except in vivo studies which represent a single study.

## Supplementary Information

**Additional file 1: Supplementary Figure S1.** Lipogenic gene expression is associated with resistance to MAPKi therapy.  **Supplementary Figure S2.** FASN and MAPK inhibition affect lipogenesis and lipid uptake. **Supplementary Figure S3.** Lipogenesis inhibition cooperates with MAPK inhibition to reduce cell proliferation. **Supplementary Figure S4.** ACACA and FASN knockdown cooperate with MAPK inhibition to reduce cell proliferation. **Supplementary Figure S5.** Lipogenesis inhibition cooperates with MAPK inhibition to reduce cell proliferation in BRAF wild-type cells. **Supplementary Figure S6.** MAPK inhibition response in Mel006 tumors is associated with membrane lipid polyunsaturation. **Supplementary Figure S7.** MAPK inhibition and FASN inhibition drive membrane polyunsaturation in D10 BMR cells. **Supplementary Figure S8.** MAPK inhibition and FASN inhibition increase membrane disorder in 451lu R and D10 BMR cells. **Supplementary Figure S9.** Phospholipidomics alterations following FASN and MAPK inhibition can be modulated by exogenous fatty acid supplementation in 451lu R. **Supplementary Figure S10.** Proliferation following FASN and MAPK inhibition can be modulated by exogenous fatty acid supplementation in D10 BMR cells. **Supplementary Figure S11.** Piperazine erastin and RSL3 sensitize D10 BMR cells to FASN and MAPK inhibition. **Supplementary Figure S12.** A host of ROS elevating compounds sensitize D10 BMR cells to FASN and MAPK inhibition. **Supplementary Figure S13.** Treatment combinations do not significantly alter mitochondrial membrane potential in 451lu R D10 BMR cells. **Supplementary Figure S14.** Exogenous fatty acid supplementation modulates the sensitivity of D10 BMR cells to FASN and MAPK inhibition in combination with ATO exposure. **Supplementary Figure S15.** Ferrostatin-1 but not ZVAD modulates the sensitivity of D10 BMR cells to FASN and MAPK inhibition in combination with ATO exposure. **Supplementary Figure S16.** The combination of FASN and MAPK inhibition with ATO treatment increases progression-free survival in Mel006 tumor bearing mice.

**Additional file 2. Mouse pathology report**

**Additional file 3. Unedited Western Blots**

## Data Availability

Data generated or analysed during this study are included in this published article and its supplementary information files and are available from the institutional repository or the corresponding author upon request.
