# Supplementary material for: Pharmacological induction of membrane lipid poly-unsaturation sensitizes melanoma to ROS inducers and overcomes acquired resistance to targeted therapy
Source: J Exp Clin Cancer Res. 2023 Apr 19;42:92. doi: 10.1186/s13046-023-02664-7 (PMC10114329; doi:10.1186/s13046-023-02664-7)
Supplement: Supplementary file 1 — Additional file 1: Supplementary Figure S1. Lipogenic gene expression is associated with resistance to MAPKi therapy. Supplementary Figure S2. FASN and MAPK inhibition affect lipogenesis and lipid uptake. Supplementary Figure S3. Lipogenesis inhibition cooperates with MAPK inhibition to reduce cell proliferation. Supplementary Figure S4. ACACA and FASN knockdown cooperate with MAPK inhibition to reduce cell proliferation. Supplementary Figure S5. Lipogenesis inhibition cooperates with MAPK inhibition to reduce cell proliferation in BRAF wild-type cells. Supplementary Figure S6. MAPK inhibition response in Mel006 tumors is associated with membrane lipid polyunsaturation. Supplementary Figure S7. MAPK inhibition and FASN inhibition drive membrane polyunsaturation in D10 BMR cells. Supplementary Figure S8. MAPK inhibition and FASN inhibition increase membrane disorder in 451lu R and D10 BMR cells. Supplementary Figure S9. Phospholipidomics alterations following FASN and MAPK inhibition can be modulated by exogenous fatty acid supplementation in 451lu R. Supplementary Figure S10. Proliferation following FASN and MAPK inhibition can be modulated by exogenous fatty acid supplementation in D10 BMR cells. Supplementary Figure S11. Piperazine erastin and RSL3 sensitize D10 BMR cells to FASN and MAPK inhibition. Supplementary Figure S12. A host of ROS elevating compounds sensitize D10 BMR cells to FASN and MAPK inhibition. Supplementary Figure S13. Treatment combinations do not significantly alter mitochondrial membrane potential in 451lu R D10 BMR cells. Supplementary Figure S14. Exogenous fatty acid supplementation modulates the sensitivity of D10 BMR cells to FASN and MAPK inhibition in combination with ATO exposure. Supplementary Figure S15. Ferrostatin-1 but not ZVAD modulates the sensitivity of D10 BMR cells to FASN and MAPK inhibition in combination with ATO exposure. Supplementary Figure S16. The combination of FASN and MAPK inhibition with ATO treatment increases pro [file 13046_2023_2664_MOESM1_ESM.pdf]

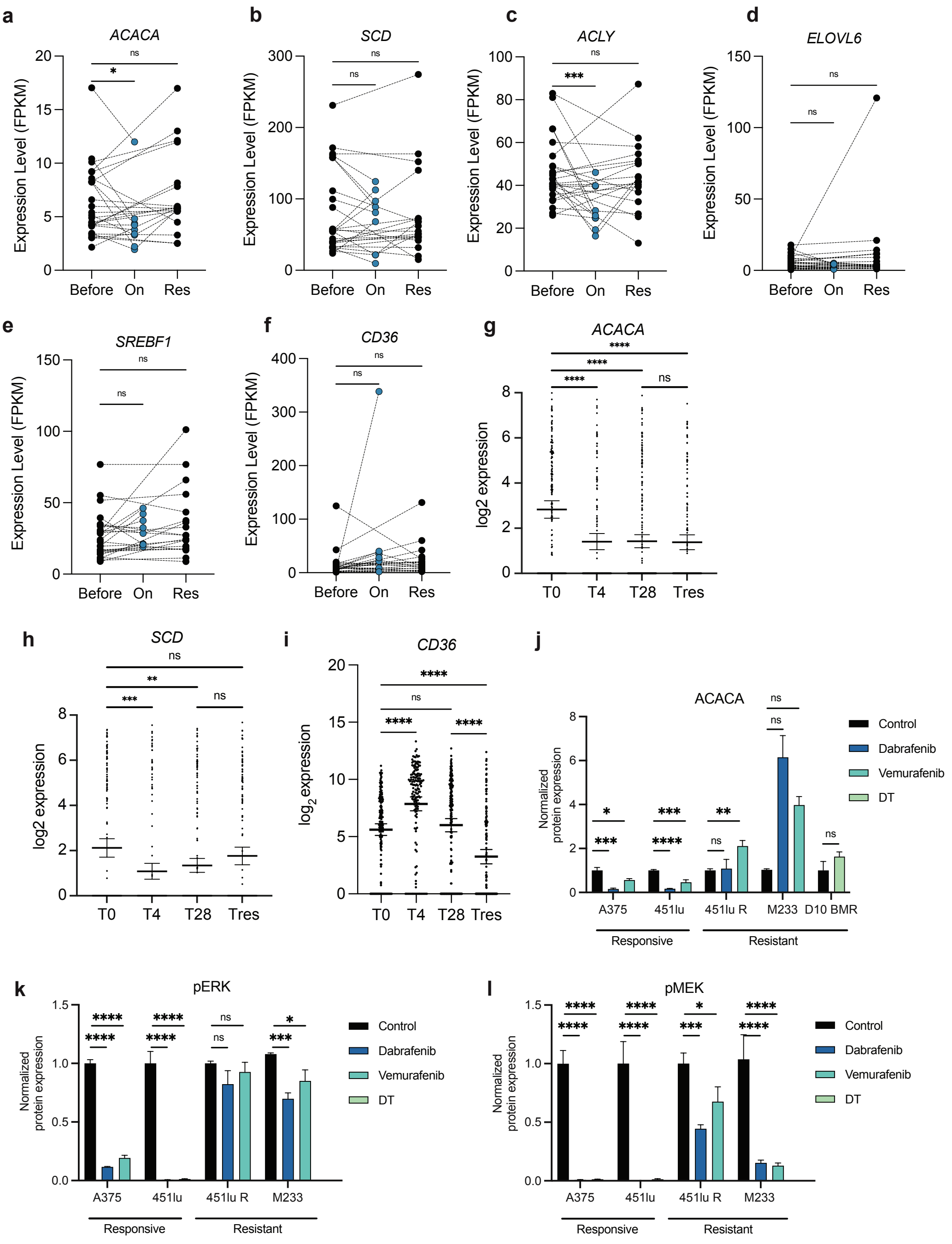

Supplementary Figure S1. Lipogenic gene expression is associated with resistance to MAPKi therapy. ACACA (**a**), SCD (**b**), ACLY (**c**), ELOVL6 (**d**), SREBF1 (**e**) and CD36 (**f**) gene expression (RNAseq) in treatment naïve (Before), on treatment (On) and in resistant clinical tumors (Res) following MAPKi. Mixed-effects analysis**.** Single-cell RNA-seq analysis of ACACA (**g**), SCD (**h**) and CD36 (**i**) expression in Mel006 PDX tumors, at T0, T4, T28 and Tres. One-way ANOVA with Tukey's multiple comparisons. Relative protein expression (western blotting) of ACACA **(j)**, pERK 1/2 **(k)** and pMEK 1/2 **(l)** in A375, 451lu, 451lu R and M233 cells following MAPKi (n=3). One-way ANOVA with Tukey's multiple comparisons. Data represent mean ± SEM of biologically independent samples. (*$p < 0.05$, **$p < 0.01$, ***$p < 0.001$, ****$p < 0.0001$

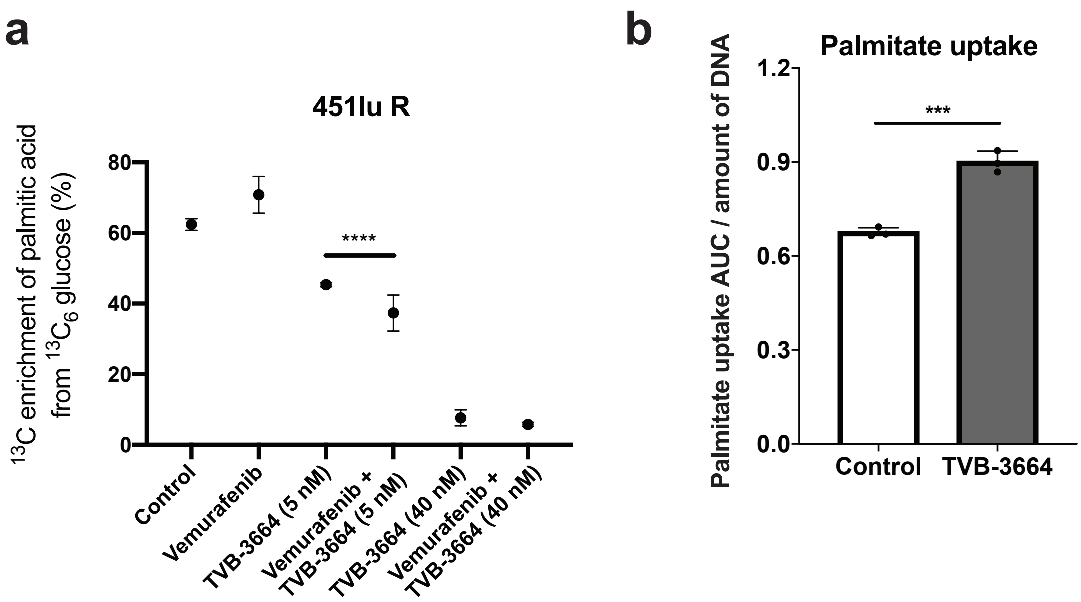

Supplementary Figure S2. FASN and MAPK inhibition affect lipogenesis and lipid uptake. **(a)** Fraction $^{13}C_6$ glucose incorporation into palmitate in 451lu R cells (n=3) following Vemurafenib and TVB-3664 treatment. One-way ANOVA with Tukey's multiple comparisons. **(b)** Palmitate media uptake as a measure of media palmitate depletion in D10 BMR cells (n=3) following TVB-3664 treatment. One-way ANOVA with Tukey's multiple comparisons. Unpaired two-sided Student's t-tests. Data represent mean ± SEM of biologically independent samples. (****p* < 0.001, *****p* < 0.0001)

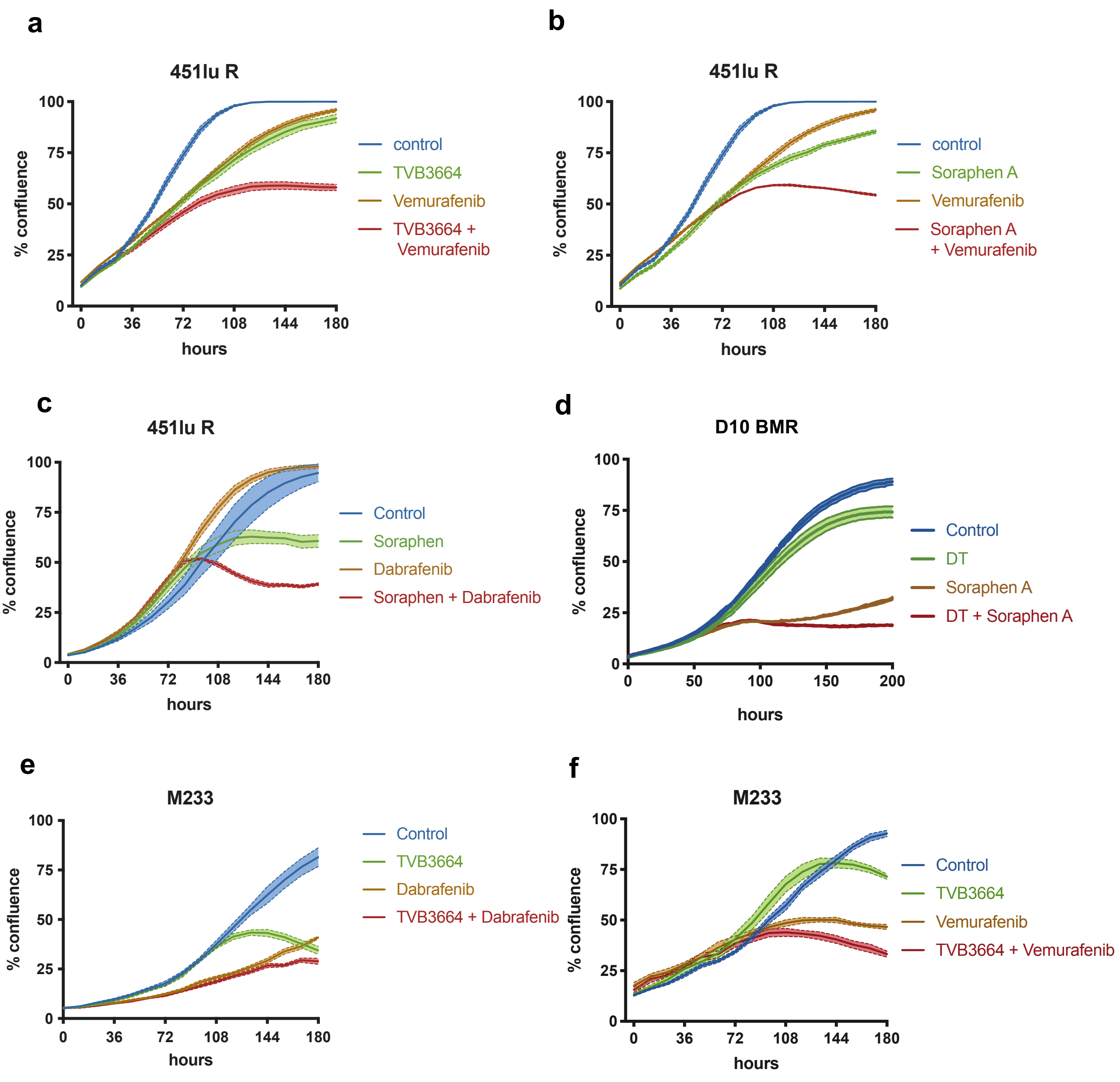

Supplementary Figure S3. Lipogenesis inhibition cooperates with MAPK inhibition to reduce cell proliferation. Real time cell growth analysis of 451lu R cells (n=6) treated with vemurafenib and **(a)** TVB-3664, or **(b)** Soraphen A, or dabrafenib and **(c)** soraphen A. Real time cell growth analysis of D10 BMR cells (n=3) following dabrafenib + trametinib and sorpahen A treatment **(d)**. Real time cell growth analysis of M233 cells (n=6) treated with dabrafenib **(e)** or vemurafenib (n=3) **(f)** and TVB-3664 (n=6). Data represent mean ± SEM of biologically independent samples.

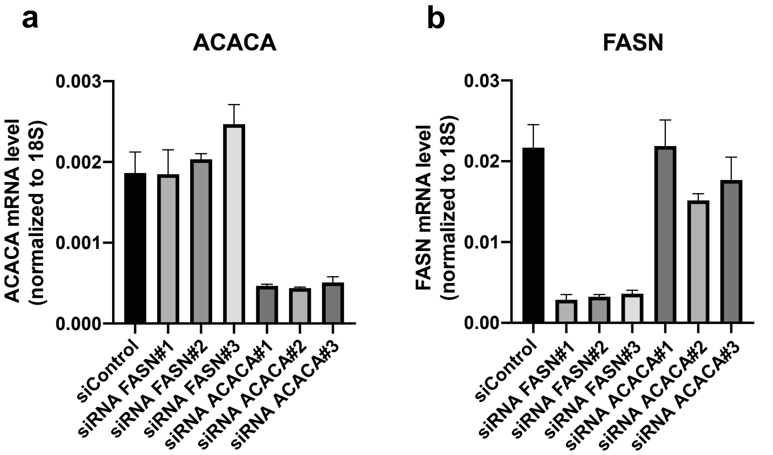

c

siRNA	sequence
siRNA FASN#1	GGUAUGCGACGGGAAAGUAtt
siRNA FASN#2	CCGUGGACCUGAUCAUCAAtt
siRNA FASN#3	GGAGCGUAUCUGUGAGAAAtt
siRNA ACACA#1	GUUUCGAAAUGAACGUGCAtt
siRNA ACACA#2	CCGGAAAUCUGAACGGGAAtt
siRNA ACACA#3	GGCUGUAUCCAUUACGUCAtt

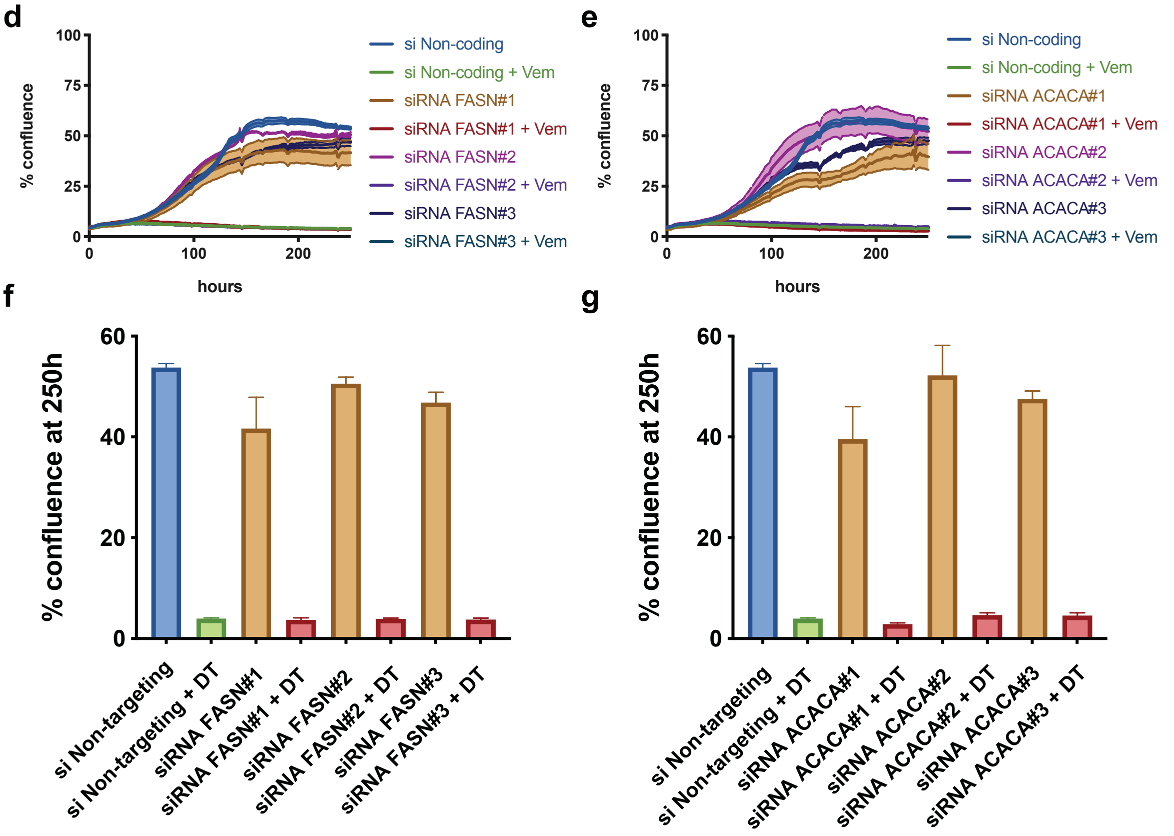

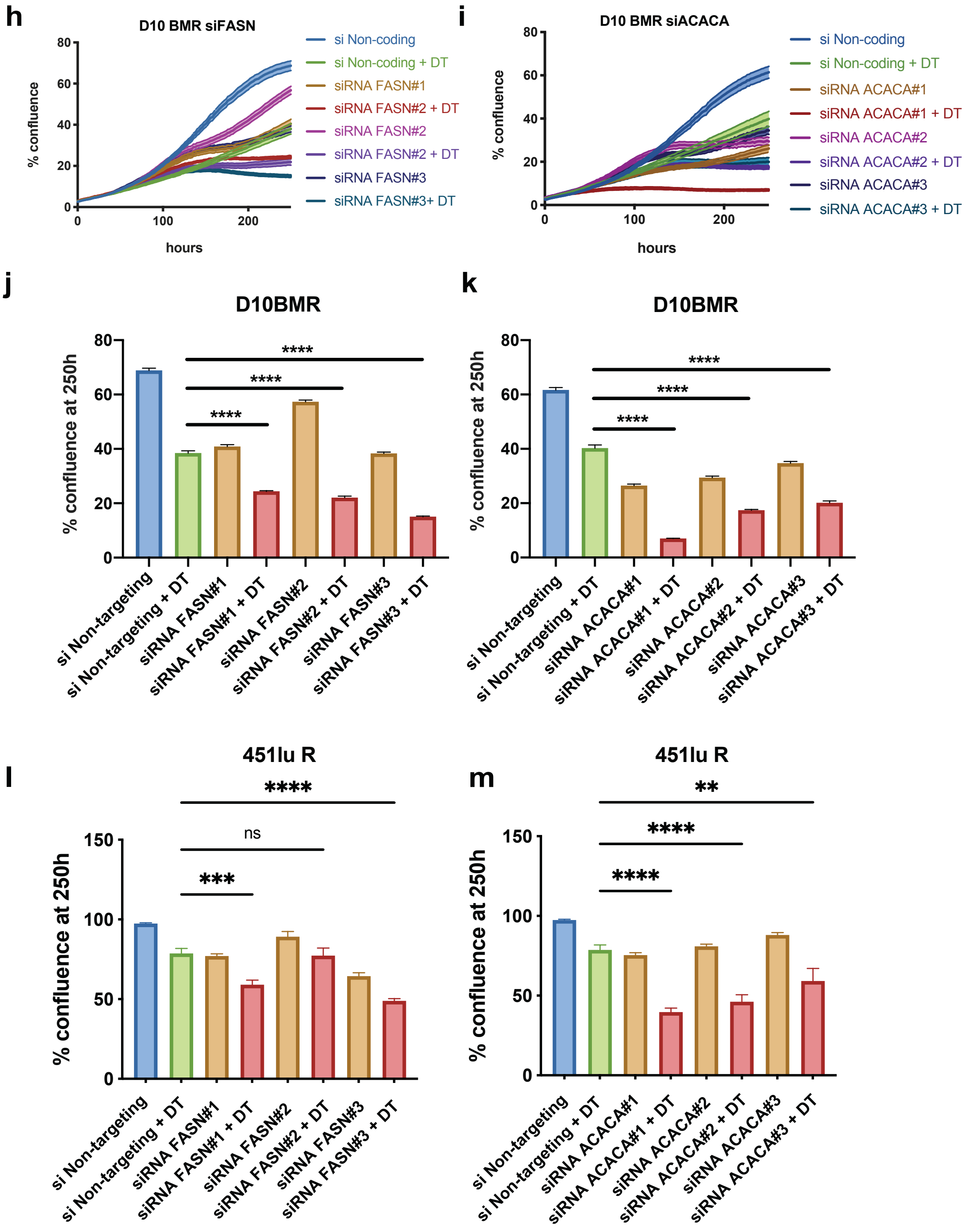

Supplementary Figure S4. ACACA and FASN knockdown cooperate with MAPK inhibition to reduce cell proliferation. Relative expression of ACACA (**a**) and FASN (**b**) in D10 BMR cells (n=3) following lipofection with siRNA constructs targeting ACACA and FASN **(c)**. Real time cell growth analysis of 451lu cells (n=3) following vemurafenib (Vem) treatment and siRNA mediated knockdown of **(d and f)** FASN and **(e and g)** ACACA. Real time cell growth analysis of D10 BMR cells (n=3) following DT treatment and siRNA mediated knockdown of **(h)** FASN and **(i)** ACACA. Real time cell growth analysis of 451lu R cells (n=3) following vemurafenib treatment and siRNA mediated knockdown of **(j and l)** FASN and **(k and m)** ACACA. One-way ANOVA with Tukey's multiple comparisons. Data represent mean ± SEM of biologically independent samples. (**$p < 0.01$, ***$p < 0.001$, ****$p < 0.0001$)

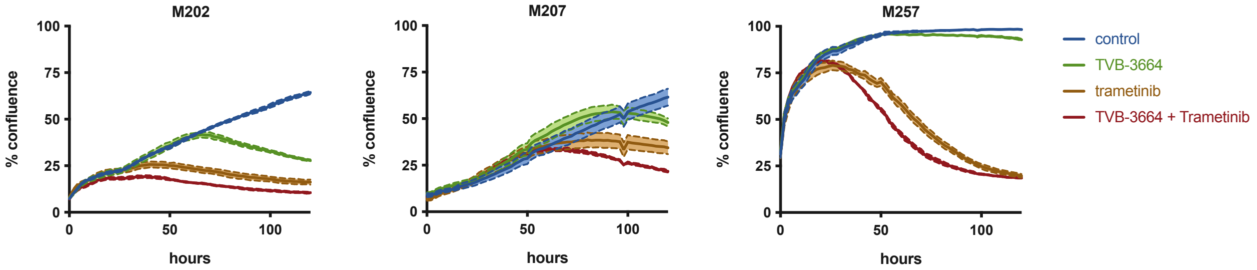

Supplementary Figure S5. Lipogenesis inhibition cooperates with MAPK inhibition to reduce cell proliferation in BRAF wild-type cells. Real time cell growth analysis of M202, M207 and M257 cells (n=3) following dabrafenib treatment in combination with TVB-3664. One-way ANOVA with Tukey's multiple comparisons. Data represent mean ± SEM of biologically independent samples. (**$p < 0.01$, ***$p < 0.001$, ****$p < 0.0001$)

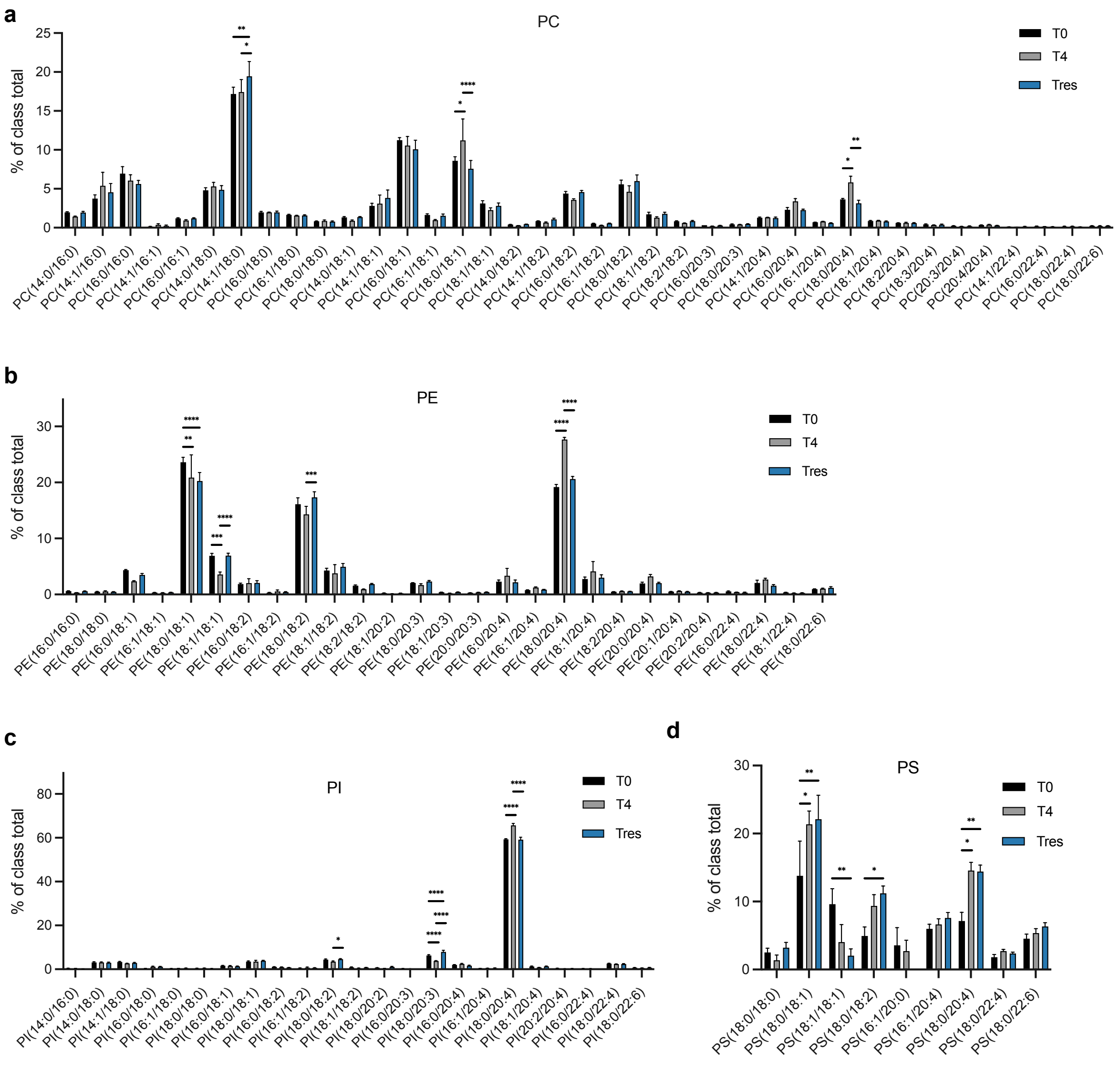

Supplementary Figure S6. MAPK inhibition response in Mel006 tumors is associated with membrane lipid polyunsaturation. Phospholipidomics of the top 95% most abundant PC (**a**) PE (**b**), PI (**c**) and PS (**d**) species in Mel006 tumors (n=6) at T0, T4 and Tres. Two-way ANOVA with Tukey's multiple comparisons. Data represent mean ± SEM of biologically independent samples. (*$p < 0.05$, **$p < 0.01$, ***$p < 0.001$, ****$p < 0.0001$)

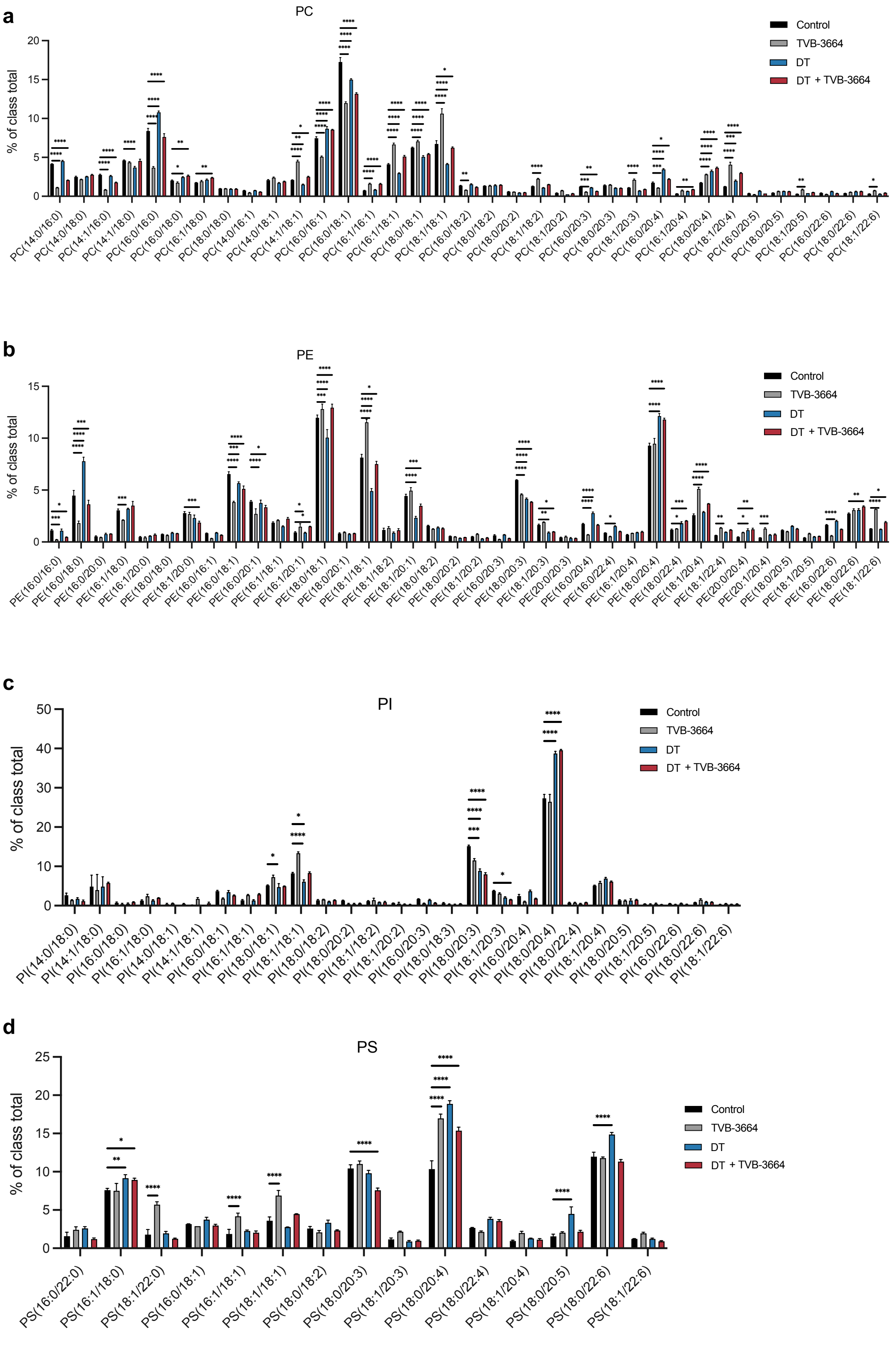

Supplementary Figure S7. MAPK inhibition and FASN inhibition drive membrane polyunsaturation in D10 BMR cells. Phospholipidomics of the top 95% most abundant PC (**a**) PE (**b**), PI (**c**) and PS (**d**) species in Mel006 tumors treated with TVB-3664 and/or Dabrafenib + Trametinib (n=3). Two-way ANOVA with Tukey's multiple comparisons. Data

represent mean ± SEM of biologically independent samples. (*$p < 0.05$, **$p < 0.01$, ***$p < 0.001$, ****$p < 0.0001$)

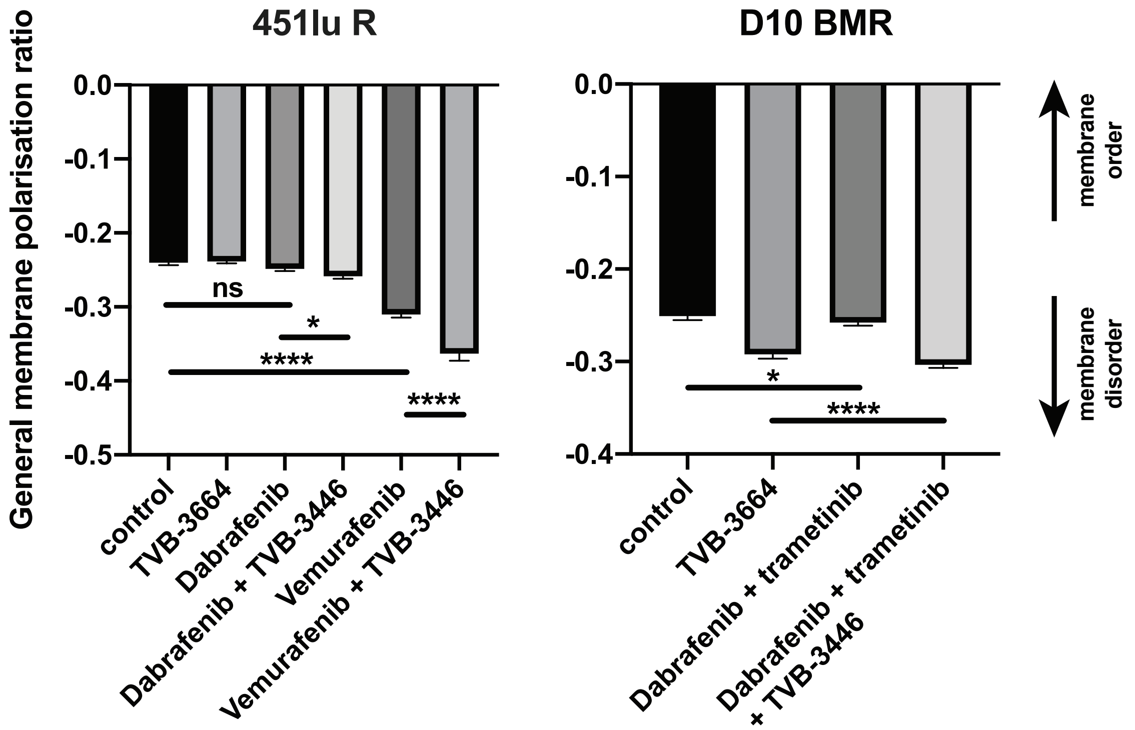

Supplementary Figure S8. MAPK inhibition and FASN inhibition increase membrane disorder in 451lu R and D10 BMR cells. Membrane order analysis of 451lu R and D10 BMR cells by Di-4-ANEPPDHQ staining under MAPKi and FASN inhibition (n=3). Two-way ANOVA with Tukey's multiple comparisons. Data represent mean ± SEM of biologically independent samples. (*$p < 0.05$, **$p < 0.01$, ***$p < 0.001$, ****$p < 0.0001$)

a

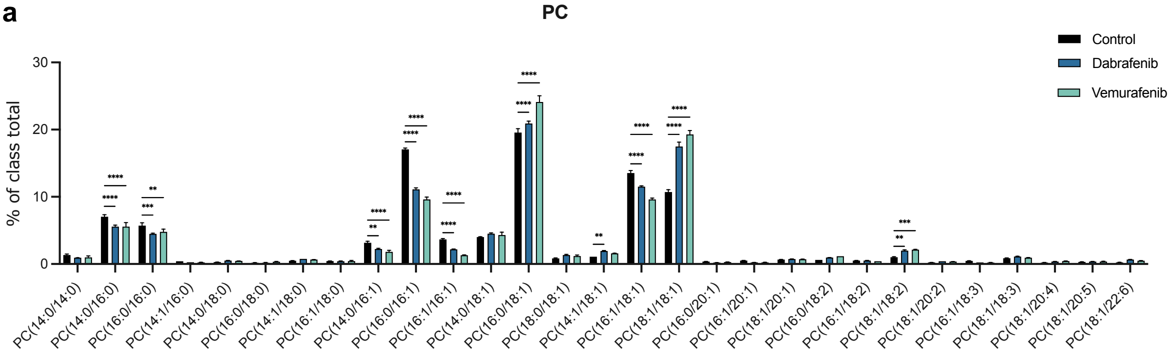
PC
Control
Dabrafenib
Vemurafenib
% of class total
PC(14:0/14:0)
PC(14:0/16:0)
PC(16:0/16:0)
PC(14:1/16:0)
PC(14:0/18:0)
PC(16:0/18:0)
PC(14:1/18:0)
PC(16:1/18:0)
PC(14:0/16:1)
PC(16:0/16:1)
PC(16:1/16:1)
PC(14:0/18:1)
PC(16:0/18:1)
PC(18:0/18:1)
PC(14:1/18:1)
PC(16:1/18:1)
PC(18:1/18:1)
PC(16:0/20:1)
PC(16:1/20:1)
PC(18:1/20:1)
PC(16:0/18:2)
PC(16:1/18:2)
PC(18:1/18:2)
PC(18:1/20:2)
PC(16:1/18:3)
PC(18:1/18:3)
PC(18:1/20:4)
PC(18:1/20:5)
PC(18:1/22:6)

b

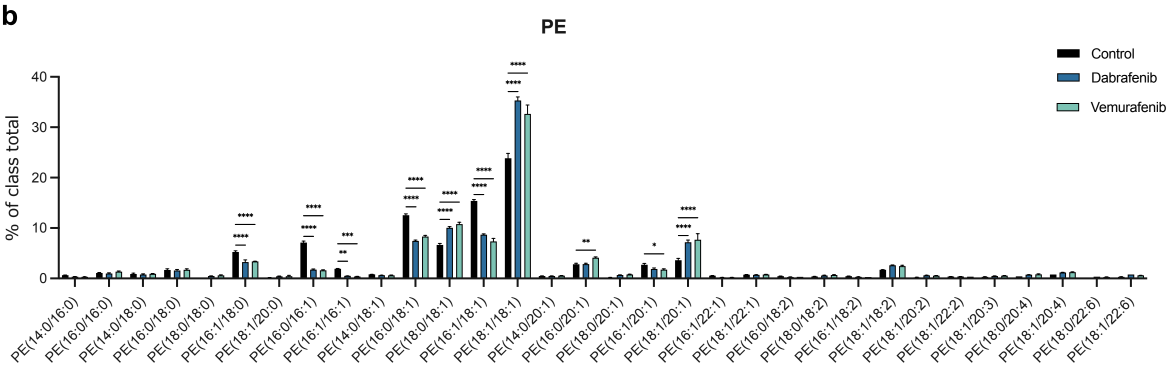
PE
Control
Dabrafenib
Vemurafenib
% of class total
PE(14:0/16:0)
PE(16:0/16:0)
PE(14:0/18:0)
PE(16:0/18:0)
PE(18:0/18:0)
PE(16:1/18:0)
PE(18:1/20:0)
PE(16:0/16:1)
PE(16:1/16:1)
PE(14:0/18:1)
PE(16:0/18:1)
PE(18:0/18:1)
PE(16:1/18:1)
PE(18:1/18:1)
PE(14:0/20:1)
PE(16:0/20:1)
PE(18:0/20:1)
PE(16:1/20:1)
PE(18:1/20:1)
PE(16:1/22:1)
PE(18:1/22:1)
PE(16:0/18:2)
PE(18:0/18:2)
PE(16:1/18:2)
PE(18:1/18:2)
PE(18:1/20:2)
PE(18:1/22:2)
PE(18:1/20:3)
PE(18:0/20:4)
PE(18:1/20:4)
PE(18:0/22:6)
PE(18:1/22:6)

c

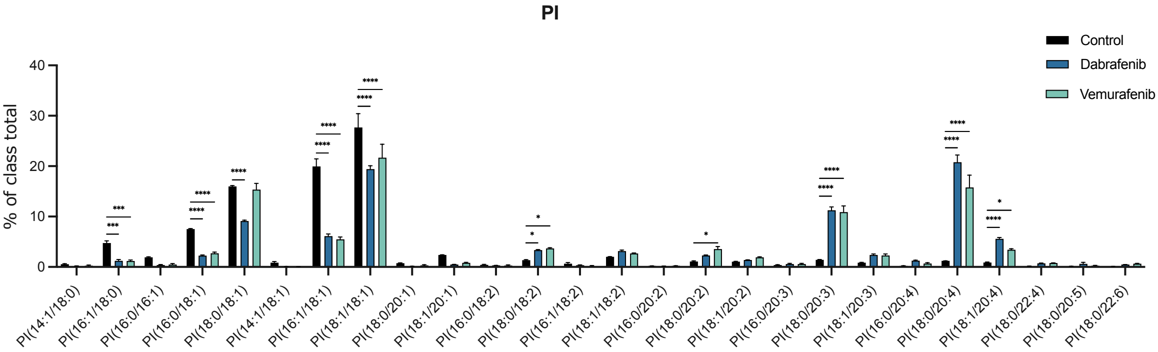
PI
Control
Dabrafenib
Vemurafenib
% of class total
PI(14:1/18:0)
PI(16:1/18:0)
PI(16:0/16:1)
PI(16:0/18:1)
PI(18:0/18:1)
PI(14:1/18:1)
PI(16:1/18:1)
PI(18:1/18:1)
PI(18:0/20:1)
PI(18:1/20:1)
PI(16:0/18:2)
PI(18:0/18:2)
PI(16:1/18:2)
PI(18:1/18:2)
PI(16:0/20:2)
PI(18:0/20:2)
PI(18:1/20:2)
PI(16:0/20:3)
PI(18:0/20:3)
PI(18:1/20:3)
PI(16:0/20:4)
PI(18:0/20:4)
PI(18:1/20:4)
PI(18:0/22:4)
PI(18:0/20:5)
PI(18:0/22:6)

d

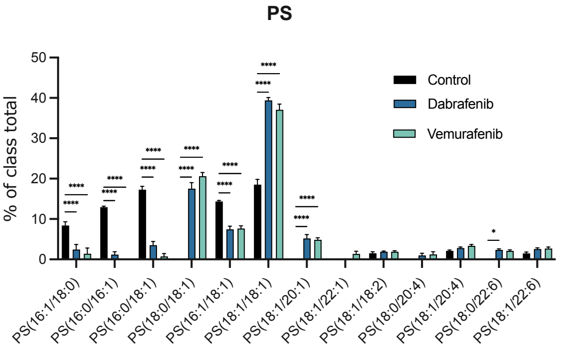
PS
Control
Dabrafenib
Vemurafenib
% of class total
PS(16:1/18:0)
PS(16:0/16:1)
PS(16:0/18:1)
PS(18:0/18:1)
PS(16:1/18:1)
PS(18:1/18:1)
PS(18:1/20:1)
PS(18:1/22:1)
PS(18:1/18:2)
PS(18:0/20:4)
PS(18:1/20:4)
PS(18:0/22:6)
PS(18:1/22:6)

e

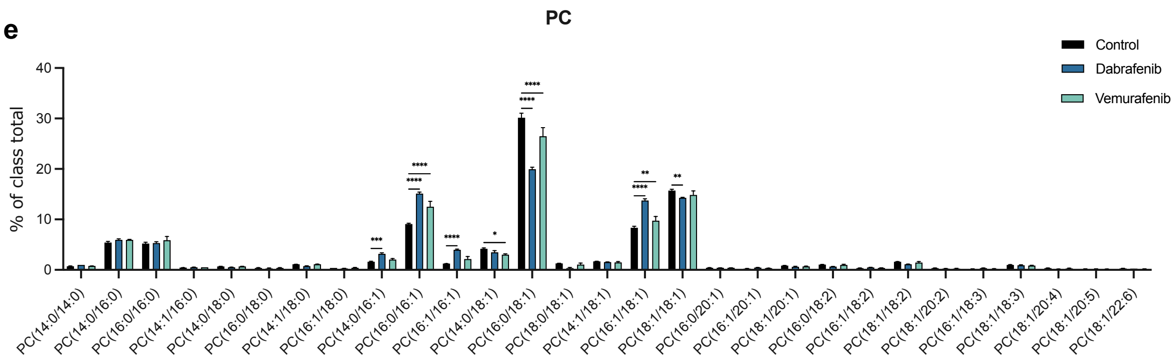
PC
Control
Dabrafenib
Vemurafenib
% of class total
0
10
20
30
40
PC(14:0/14:0)
PC(14:0/16:0)
PC(16:0/16:0)
PC(14:1/16:0)
PC(14:0/18:0)
PC(16:0/18:0)
PC(14:1/18:0)
PC(16:1/18:0)
PC(14:0/16:1)
PC(16:0/16:1)
PC(16:1/16:1)
PC(14:0/18:1)
PC(16:0/18:1)
PC(18:0/18:1)
PC(14:1/18:1)
PC(16:1/18:1)
PC(18:1/18:1)
PC(16:0/20:1)
PC(16:1/20:1)
PC(18:1/20:1)
PC(16:0/18:2)
PC(16:1/18:2)
PC(18:1/18:2)
PC(18:1/20:2)
PC(16:1/18:3)
PC(18:1/18:3)
PC(18:1/20:4)
PC(18:1/20:5)
PC(18:1/22:6)

f

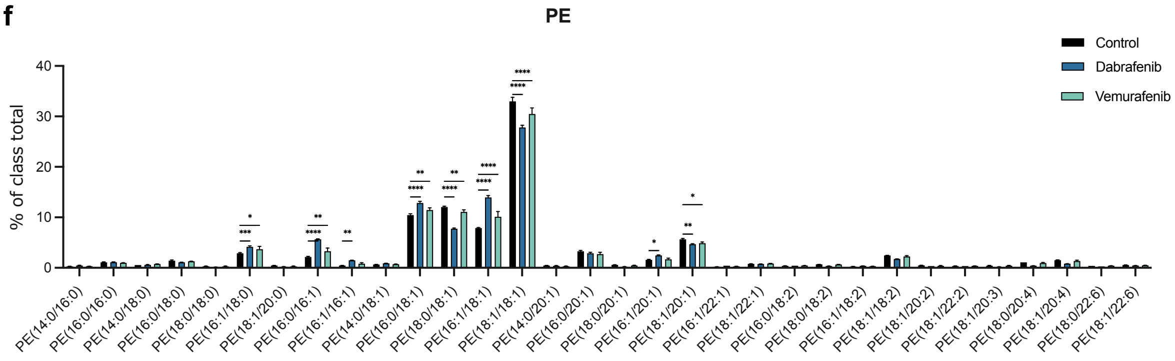
PE
Control
Dabrafenib
Vemurafenib
% of class total
0
10
20
30
40
PE(14:0/16:0)
PE(16:0/16:0)
PE(14:0/18:0)
PE(16:0/18:0)
PE(18:0/18:0)
PE(16:1/18:0)
PE(18:1/20:0)
PE(16:0/16:1)
PE(16:1/16:1)
PE(14:0/18:1)
PE(16:0/18:1)
PE(18:0/18:1)
PE(16:1/18:1)
PE(18:1/18:1)
PE(14:0/20:1)
PE(16:0/20:1)
PE(18:0/20:1)
PE(16:1/20:1)
PE(18:1/20:1)
PE(16:1/22:1)
PE(18:1/22:1)
PE(16:0/18:2)
PE(18:0/18:2)
PE(16:1/18:2)
PE(18:1/18:2)
PE(18:1/20:2)
PE(18:1/22:2)
PE(18:1/20:3)
PE(18:0/20:4)
PE(18:1/20:4)
PE(18:0/22:6)
PE(18:1/22:6)

g

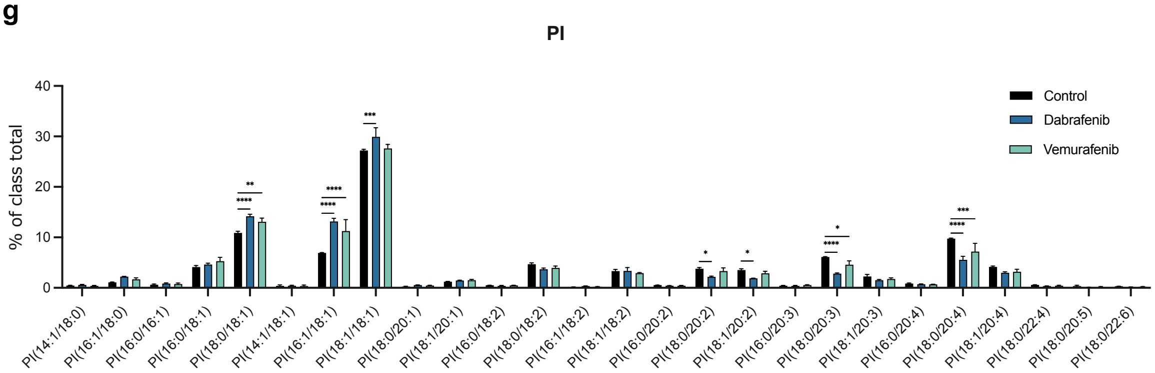
PI
Control
Dabrafenib
Vemurafenib
% of class total
0
10
20
30
40
PI(14:1/18:0)
PI(16:1/18:0)
PI(16:0/16:1)
PI(16:0/18:1)
PI(18:0/18:1)
PI(14:1/18:1)
PI(16:1/18:1)
PI(18:1/18:1)
PI(18:0/20:1)
PI(18:1/20:1)
PI(16:0/18:2)
PI(18:0/18:2)
PI(16:1/18:2)
PI(18:1/18:2)
PI(16:0/20:2)
PI(18:0/20:2)
PI(18:1/20:2)
PI(16:0/20:3)
PI(18:0/20:3)
PI(18:1/20:3)
PI(16:0/20:4)
PI(18:0/20:4)
PI(18:1/20:4)
PI(18:0/22:4)
PI(18:0/20:5)
PI(18:0/22:6)

h

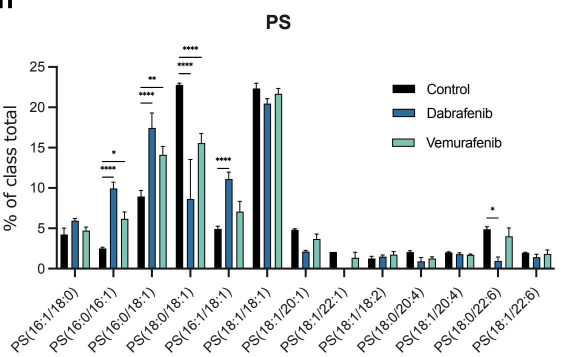
PS
Control
Dabrafenib
Vemurafenib
% of class total
0
5
10
15
20
25
PS(16:1/18:0)
PS(16:0/16:1)
PS(16:0/18:1)
PS(18:0/18:1)
PS(16:1/18:1)
PS(18:1/18:1)
PS(18:1/20:1)
PS(18:1/22:1)
PS(18:1/18:2)
PS(18:0/20:4)
PS(18:1/20:4)
PS(18:0/22:6)
PS(18:1/22:6)

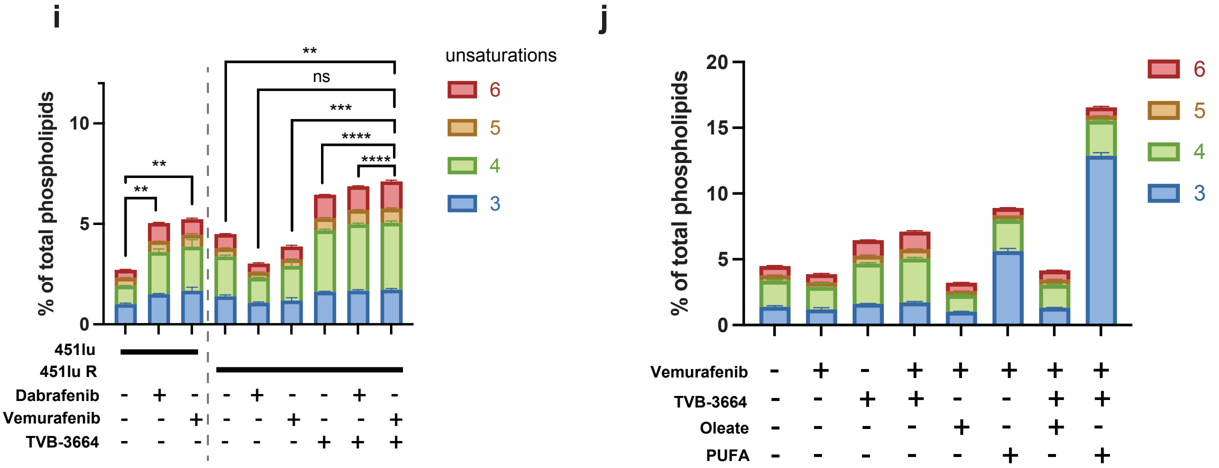

Supplementary Figure S9. Phospholipidomics alterations following FASN and MAPK inhibition can be modulated by exogenous fatty acid supplementation in 451lu R. Phospholipidomics of the top 95% most abundant PC (**a**) PE (**b**), PI (**c**) and PS (**d**) species in 451lu cells (n=3) treated with dabrafenib or vemurafenib. Two-way ANOVA with Tukey's multiple comparisons. Phospholipidomics of the top 95% most abundant PC (**e**) PE (**f**), PI (**g**) and PS (**h**) species in 451lu R cells (n=3) treated with dabrafenib or vemurafenib. Two-way ANOVA with Tukey's multiple comparisons. Saturation degree of total levels of PC, PE, PI, PS and PG in 451 lu (n=3) and 451lu R cells (n=3) upon treatment compared to baseline levels **(i and j)**. One-way ANOVA with Tukey's multiple comparisons. Data represent mean ± SEM of biologically independent samples. (*$p < 0.05$, **$p < 0.01$, ***$p < 0.001$, ****$p < 0.0001$)

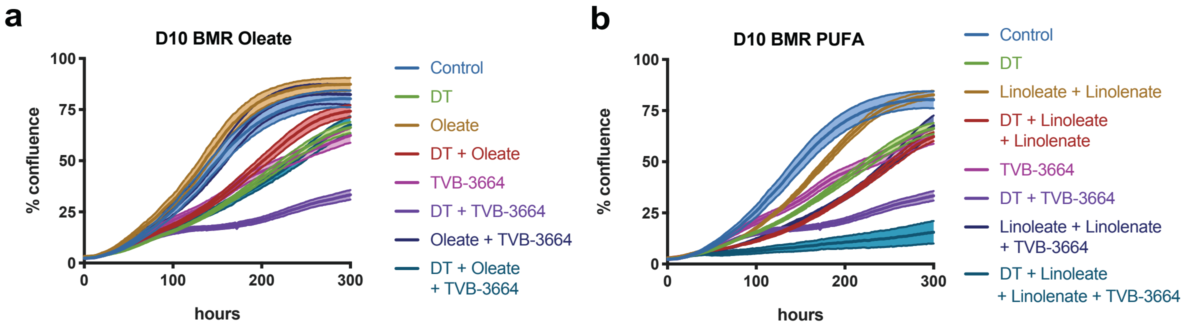

Supplementary Figure S10. Proliferation following FASN and MAPK inhibition can be modulated by exogenous fatty acid supplementation in D10 BMR cells. Real time cell growth analysis of D10 BMR cells (n=6) with supplementation of exogenous **(a)** oleate, or **(b)** a mixture of linoleate and linolenate. Data represent mean ± SEM of biologically independent samples.

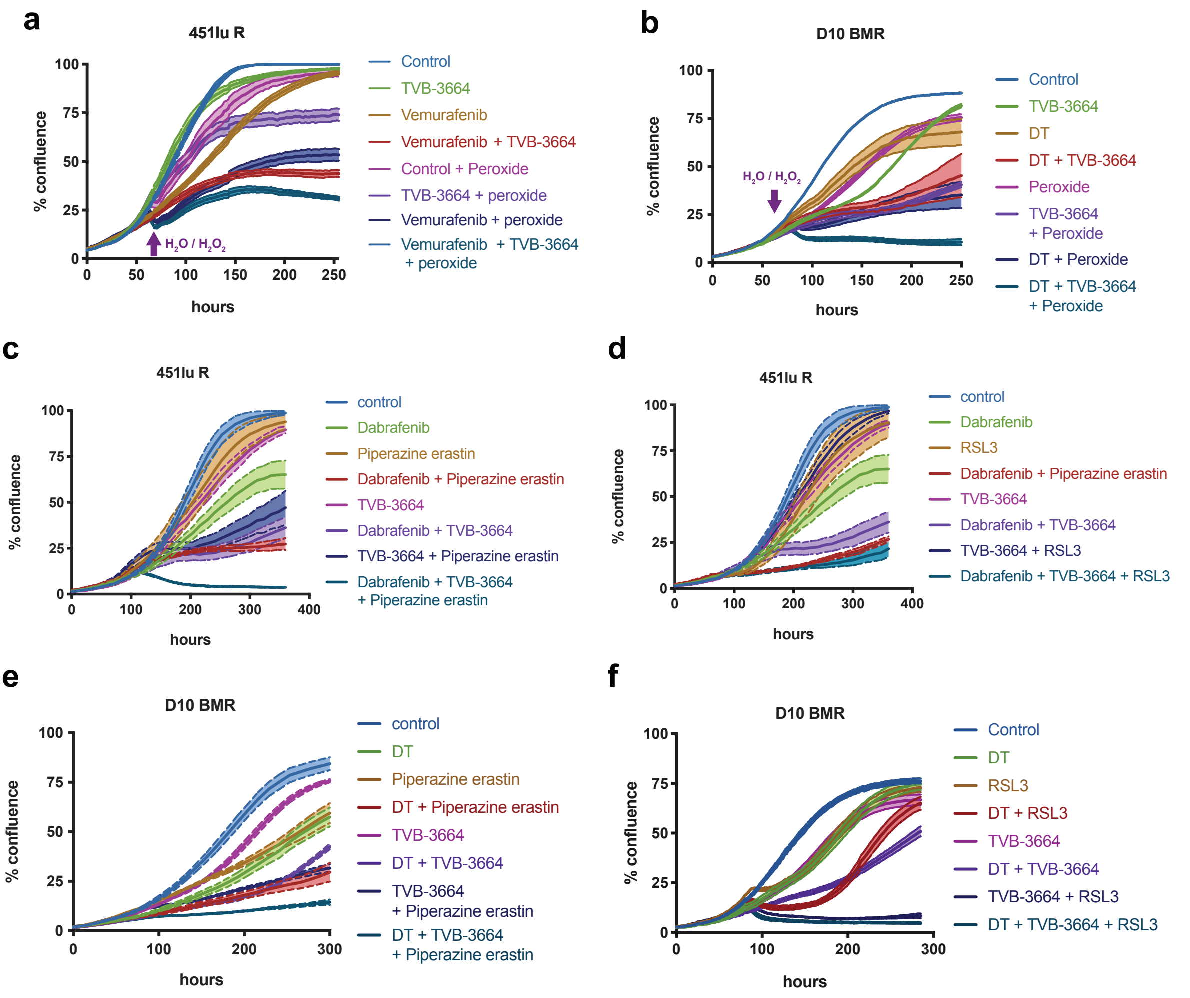

Supplementary Figure S11. Piperazine erastin and RSL3 sensitize D10 BMR cells to FASN and MAPK inhibition. Real time cell growth analysis of 451lu R cells (n=3) treated with vemurafenib, TVB-3664 in combination with **(a)** piperazine erastin or **(b)** RSL3. Data represent mean ± SEM. Representative experiment of biological triplicates. Real time cell growth analysis of 451lu R cells treated with vemurafenib, TVB-3664 in combination with **(c)** piperazine erastin or **(d)** RSL3. Real time cell growth analysis of D10BMR cells (n=3) treated with DT, TVB-3664 and **(e)** piperazine erastin or **(f)** RSL3. Data represent mean ± SEM of biologically independent samples.

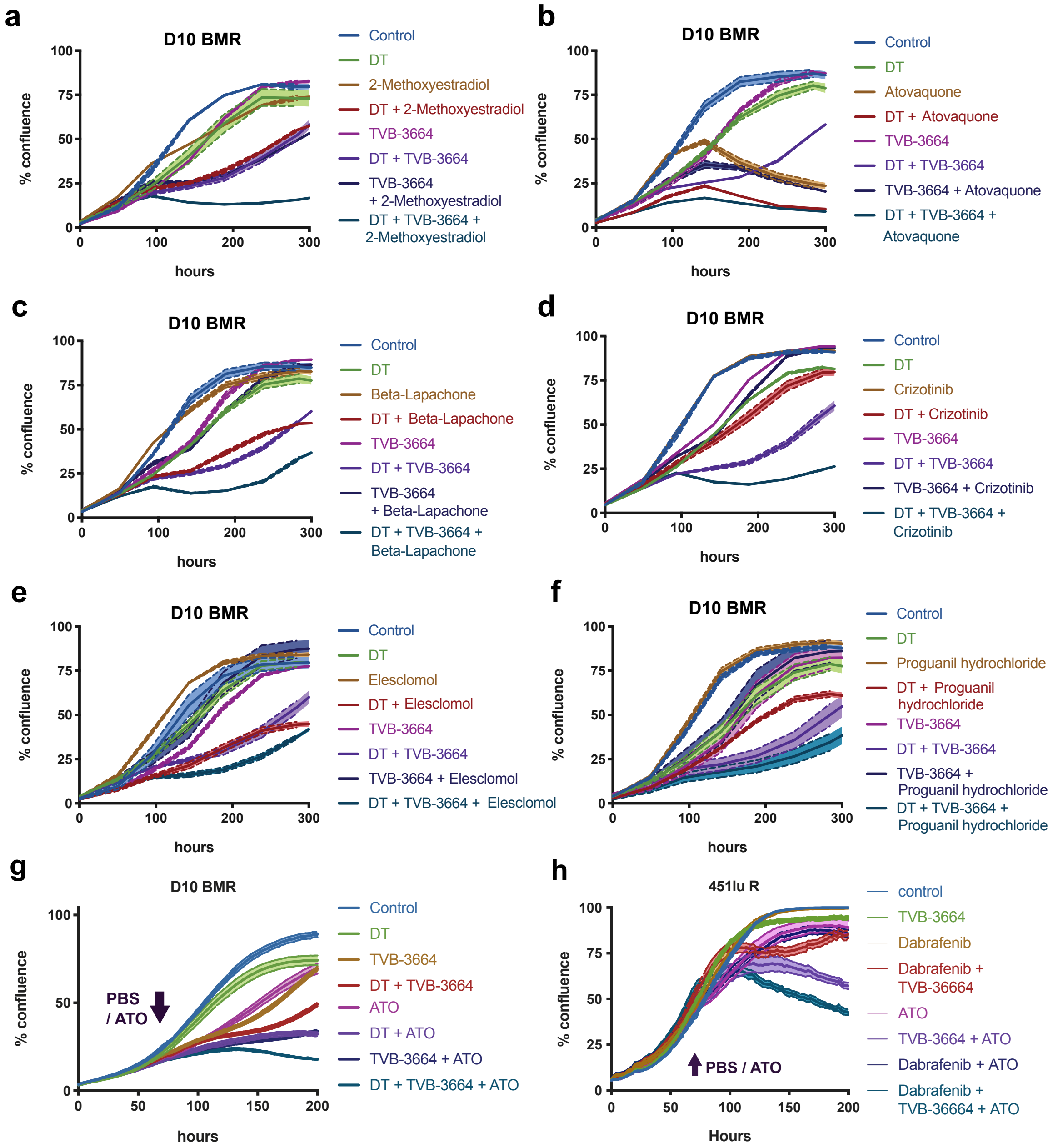

Supplementary Figure S12. A host of ROS elevating compounds sensitize D10 BMR cells to FASN and MAPK inhibition. Real time cell growth analysis of D10 BMR cells treated with DT, TVB-3664 in combination with **(a)** 2-methoxyestradiol (n=2), **(b)** atovaquone (n=2), **(c)** beta-lapachone (n=2), **(d)** crizotinib (n=2), **(e)** elesclomol (n=2), **(f)** proguanil hydrochloride (n=3) or **(g)** ATO (n=3). **h** Real time cell growth analysis of 451lu R cells (n=3) treated with dabrafenib, TVB-3664 in combination with ATO. Data represent mean ± SEM of biologically independent samples.

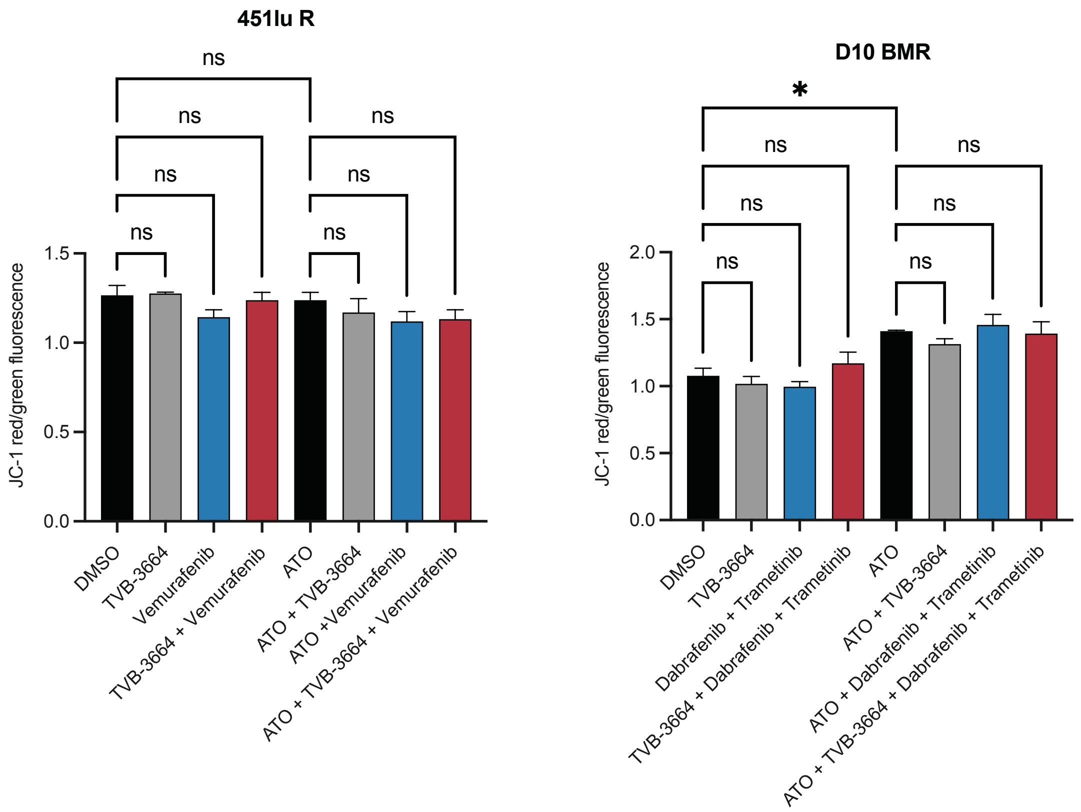

Supplementary Figure S13. Treatment combinations do not significantly alter mitochondrial membrane potential in 451lu R D10 BMR cells. JC-1 staining as a surrogate of mitochondrial membrane potential in 451lu R and D10 BMR cells (n=3) treated with MAPKi, ATO and TVB-3664 at 120 hours. Two-way ANOVA with Tukey's multiple comparisons. Data represent mean ± SEM of biologically independent samples. (*$p < 0.05$, ***$p < 0.001$, ****$p < 0.0001$)

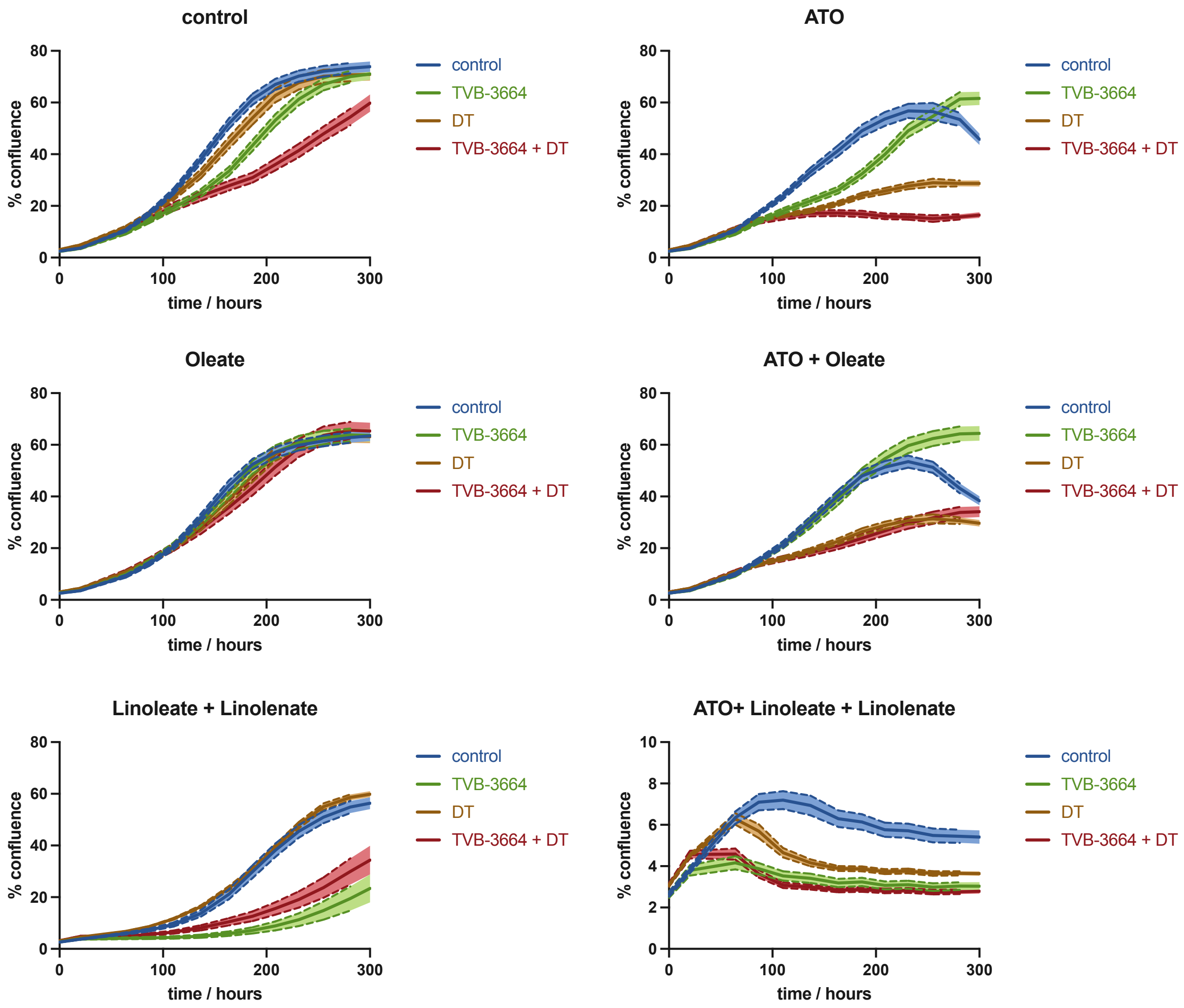

Supplementary Figure S14. Exogenous fatty acid supplementation modulates the sensitivity of D10 BMR cells to FASN and MAPK inhibition in combination with ATO exposure. Real time cell growth analysis of D10 BMR cells (n=3) treated with DT, TVB-3664 and ATO with oleate or a mixture of linoleate and linolenate (PUFA). Data represent mean ± SEM of biologically independent samples.

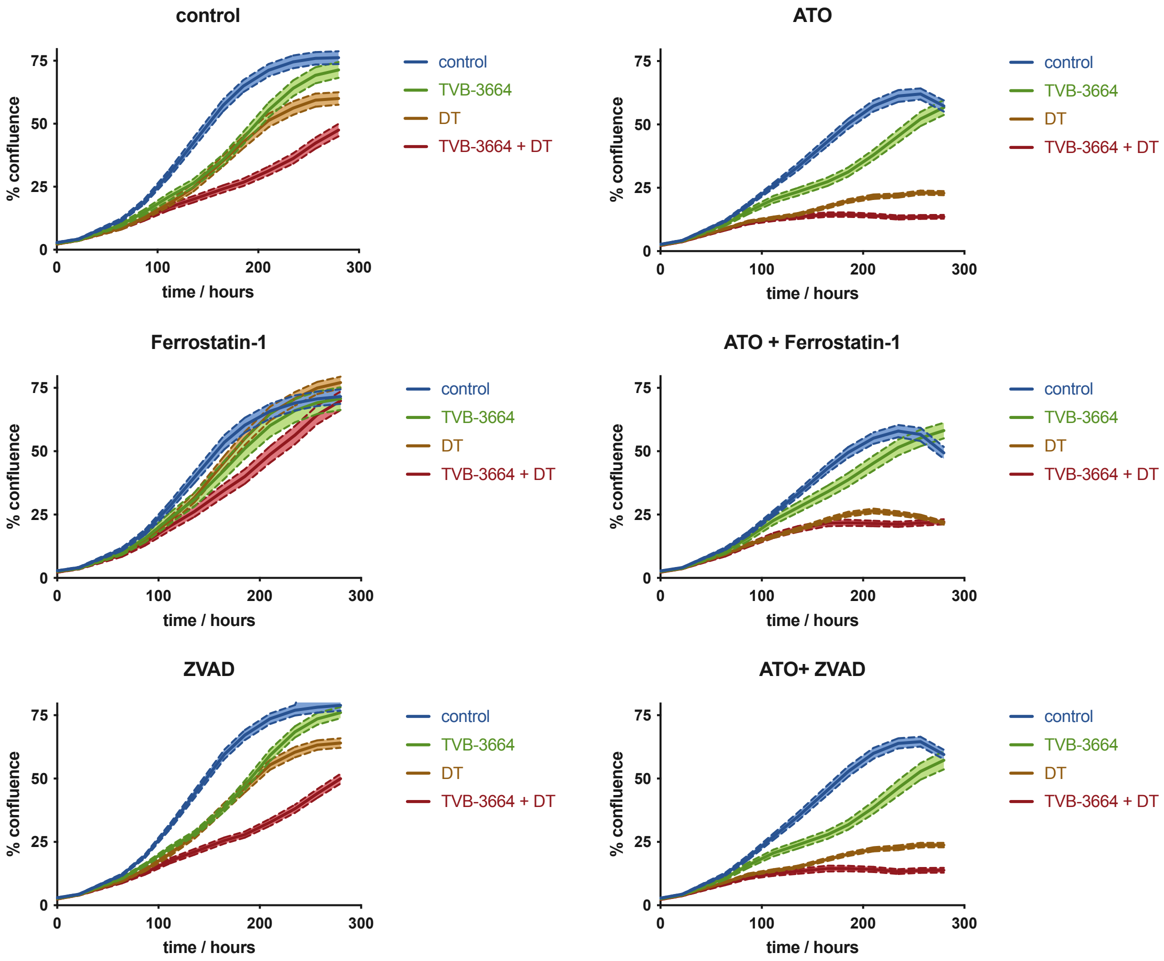

Supplementary Figure S15. Ferrostatin-1 but not ZVAD modulates the sensitivity of D10 BMR cells to FASN and MAPK inhibition in combination with ATO exposure. Real time cell growth analysis of D10 BMR cells (n=3) treated with DT, TVB-3664 and ATO with ferrostatin-1 or ZVAD. Data represent mean ± SEM of biologically independent samples.

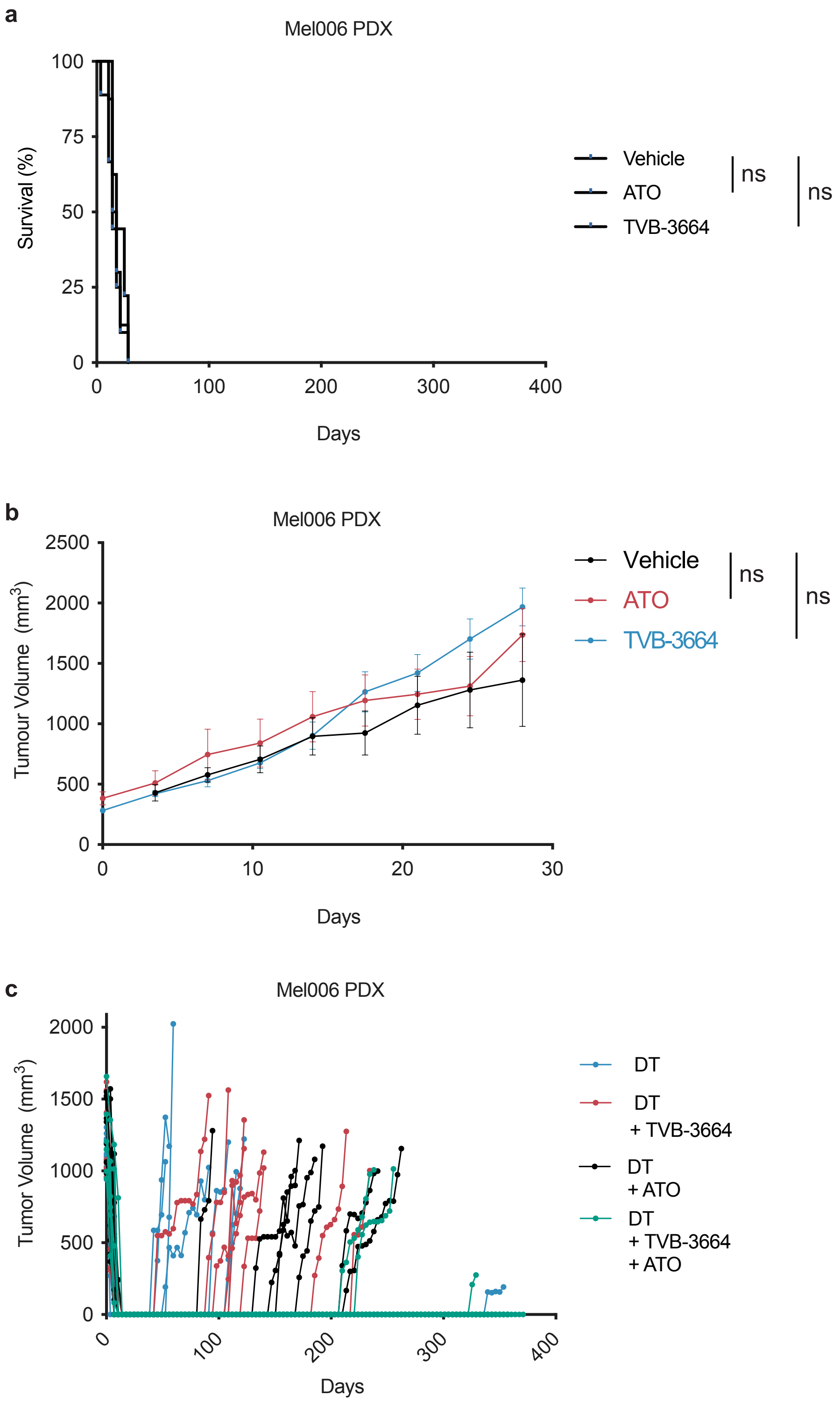

Supplementary Figure S16. The combination of FASN and MAPK inhibition with ATO treatment increases progression-free survival in Mel006 tumor bearing mice. (a) Survival of Mel006 PDX tumor bearing mice following treatment with vehicle (n=10), ATO (n=9) or TVB-3664 (n=8). Log-rank (Mantel-Cox) test. Data represent mean ± SEM. **(b)** Tumor growth curve of Mel006 PDX tumor bearing mice following treatment with DT (n=8), DT + TVB-3664 (n=8), DT + ATO (n=10) or DT + TVB-3664 + ATO (n=7). One-way ANOVA with Tukey's multiple comparisons. **(c)** Tumor volumes of individual Mel006 PDX tumor bearing mice following indicated treatments. Data depict individual mice.
