# Supplementary material for: Pharmacological induction of membrane lipid poly-unsaturation sensitizes melanoma to ROS inducers and overcomes acquired resistance to targeted therapy
Source: J Exp Clin Cancer Res. 2023 Apr 19;42:92. doi: 10.1186/s13046-023-02664-7 (PMC10114329; doi:10.1186/s13046-023-02664-7)
Supplement: Supplementary file 2 — Additional file 2. Mouse pathology report [file 13046_2023_2664_MOESM2_ESM.docx]

**Mice were subjected to pathology analysis blindly**

**Treatment conditions:**

- Ex7343 = Dabrafenib + trametinib

- EX7350 = Dabrafenib + trametinib

- EX7459 = Dabrafenib + trametinib + TVB-3664

- EX7467 = Dabrafenib + trametinib + TVB-3664

- EX7692 = Dabrafenib + trametinib + ATO

- EX7699 = Dabrafenib + trametinib + ATO

- EX7706 = Dabrafenib + trametinib + TVB-3664 + ATO

- EX7713 = Dabrafenib + trametinib + TVB-3664 + ATO

**Toxicology Report of mouse EX-7343**

**Gross pathology**:

Following median thoracal and abdominal section, following organs were visualized and biopsies were prelevated:

Lungs: no abnormalities on macroscopy

Heart: no abnormalities on macroscopy

Thymus: no abnormalities on macroscopy

Thoracic cavity: no abnormalities on macroscopy, no fluid

Liver: no abnormalities on macroscopy

Pancreas: no abnormalities on macroscopy

Spleen: no abnormalities on macroscopy

Stomach and bowel segments: no abnormalities on macroscopy

Kidneys: no abnormalities on macroscopy

**Microscopical examination**:

**Lungs**: large central bronchial branches show peribronchial inflammation and reactive changes of the epithelium. No viral inclusions are seen. This is a picture of **bronchitis, most probably, viral bronchitis**. The lung alveolar parenchyma show normal aeration and no inflammation, no malignancy present.

**Heart**: normal architecture, no inflammation, no malignancy

**Liver**: normal architecture, portal tracts contain bile duct, artery and vein, no inflammation. The parenchyma does not show cell drop-out, no centrolobular induction, no inflammation. No malignancy.

**Kidneys**: Normal cortical and medullar architecture, glomeruli show no abnormalities, different segments of the tubules show no signs of necrosis, inflammation or fibrosis. No interstitial lesions. No vascular lesions.

**Pancreas**: normal architecture, no inflammation, no necrosis, no malignancy

**Spleen**: normal architecture and presence of red and white pulpa, no inflammation, no bleeding. No malignancy, signs of haematopoiesis

**Tumour**: solid growing tumour consisting of large cells with atypical nuclei.

**Toxicology Report of mouse EX-7350**

**Gross pathology**:

Following median thoracal and abdominal section, following organs were visualized and biopsies were prelevated:

Lungs: no abnormalities on macroscopy

Heart: no abnormalities on macroscopy

Thymus: no abnormalities on macroscopy

Thoracic cavity: no abnormalities on macroscopy, no fluid

Liver: no abnormalities on macroscopy

Pancreas: no abnormalities on macroscopy

Spleen: no abnormalities on macroscopy

Stomach and bowel segments: no abnormalities on macroscopy

Kidneys: no abnormalities on macroscopy

**Microscopical examination**:

**Lungs**: Different branches of the bronchial tree show no abnormalities: no inflammation, no malignancy. The lung alveolar parenchyma show normal aeration and no inflammation, no malignancy present.

**Heart**: normal architecture, no inflammation, no malignancy

**Liver**: normal architecture, portal tracts contain bile duct, artery and vein, no inflammation. The parenchyma does not show cell drop-out, no centrolobular induction, no inflammation. No malignancy.

**Kidneys**: Normal cortical and medullar architecture, glomeruli show no abnormalities, different segments of the tubules show no signs of necrosis, inflammation or fibrosis. No interstitial lesions. No vascular lesions.

**Pancreas**: normal architecture, no inflammation, no necrosis, no malignancy

**Spleen**: normal architecture and presence of red and white pulpa, no inflammation, no bleeding. No malignancy, signs of haematopoiesis

**Tumour**: solid growing tumour consisting of large cells with atypical nuclei.

**Toxicology Report of mouse EX-7459**

**Gross pathology**:

Following median thoracal and abdominal section, following organs were visualized and biopsies were prelevated:

Lungs: no abnormalities on macroscopy

Heart: no abnormalities on macroscopy

Thymus: no abnormalities on macroscopy

Thoracic cavity: no abnormalities on macroscopy, no fluid

Liver: no abnormalities on macroscopy

Pancreas: no abnormalities on macroscopy

Spleen: no abnormalities on macroscopy

Stomach and bowel segments: no abnormalities on macroscopy

Kidneys: no abnormalities on macroscopy

**Microscopical examination**:

**Lungs**: Different branches of the bronchial tree show no abnormalities: no inflammation, no malignancy. The lung alveolar parenchyma show normal aeration and no inflammation, no malignancy present.

**Heart**: normal architecture, no inflammation, no malignancy

**Liver**: normal architecture, portal tracts contain bile duct, artery and vein, no inflammation. The parenchyma does not show cell drop-out, no centrolobular induction, no inflammation. No malignancy.

**Kidneys**: Normal cortical and medullar architecture, glomeruli show no abnormalities, different segments of the tubules show no signs of necrosis, inflammation or fibrosis. No interstitial lesions. No vascular lesions.

**Pancreas**: normal architecture, no inflammation, no necrosis, no malignancy

**Spleen**: normal architecture and presence of red and white pulpa, no inflammation, no bleeding. No malignancy, signs of haematopoiesis

**Tumour**: solid growing tumour consisting of large cells with atypical nuclei.

**Toxicology Report of mouse EX-7467**

**Gross pathology**:

Following median thoracal and abdominal section, following organs were visualized and biopsies were prelevated:

Lungs: no abnormalities on macroscopy

Heart: no abnormalities on macroscopy

Thymus: no abnormalities on macroscopy

Thoracic cavity: no abnormalities on macroscopy, no fluid

Liver: no abnormalities on macroscopy

Pancreas: no abnormalities on macroscopy

Spleen: no abnormalities on macroscopy

Stomach and bowel segments: no abnormalities on macroscopy

Kidneys: no abnormalities on macroscopy

**Microscopical examination**:

**Lungs**: Different branches of the bronchial tree show no abnormalities: no inflammation, no malignancy. The lung alveolar parenchyma show normal aeration and no inflammation, no malignancy present.

**Heart**: normal architecture, no inflammation, no malignancy

**Liver**: normal architecture, portal tracts contain bile duct, artery and vein, no inflammation. The parenchyma does not show cell drop-out, no centrolobular induction, no inflammation. No malignancy.

**Kidneys**: Normal cortical and medullar architecture, glomeruli show no abnormalities, different segments of the tubules show no signs of necrosis, inflammation or fibrosis. No interstitial lesions. No vascular lesions.

**Pancreas**: normal architecture, no inflammation, no necrosis, no malignancy

**Spleen**: normal architecture and presence of red and white pulpa, no inflammation, no bleeding. No malignancy, signs of haematopoiesis

**Tumour**: solid growing tumour consisting of large cells with atypical nuclei.

**Toxicology Report of mouse EX-7692**

**Gross pathology**:

Following median thoracal and abdominal section, following organs were visualized and biopsies were prelevated:

Lungs: no abnormalities on macroscopy

Heart: no abnormalities on macroscopy

Thymus: no abnormalities on macroscopy

Thoracic cavity: no abnormalities on macroscopy, no fluid

Liver: no abnormalities on macroscopy

Pancreas: no abnormalities on macroscopy

Spleen: no abnormalities on macroscopy

Stomach and bowel segments: no abnormalities on macroscopy

Kidneys: no abnormalities on macroscopy

**Microscopical examination**:

**Lungs**: Different branches of the bronchial tree show no abnormalities: no inflammation, no malignancy. The lung alveolar parenchyma show normal aeration and no inflammation, no malignancy present.

**Heart**: normal architecture, no inflammation, no malignancy

**Liver**: normal architecture, portal tracts contain bile duct, artery and vein, no inflammation. The parenchyma does not show cell drop-out, no centrolobular induction, no inflammation. No malignancy.

**Kidneys**: Normal cortical and medullar architecture, glomeruli show no abnormalities, different segments of the tubules show no signs of necrosis, inflammation or fibrosis. No interstitial lesions. No vascular lesions.

**Spleen**: normal architecture and presence of red and white pulpa, no inflammation, no bleeding. No malignancy. There are signs of extramedullary haematopoiesis and presence of some haemosiderin loaden macrophages. The latter could be due to the tumour necrosis

**Tumour**: solid growing tumour consisting of large cells with atypical nuclei. There is tumour necrosis with bleeding.

**Toxicology Report of mouse EX-7699**

**Gross pathology**:

Following median thoracal and abdominal section, following organs were visualized and biopsies were prelevated:

Lungs: no abnormalities on macroscopy

Heart: no abnormalities on macroscopy

Thymus: no abnormalities on macroscopy

Thoracic cavity: no abnormalities on macroscopy, no fluid

Liver: no abnormalities on macroscopy

Pancreas: no abnormalities on macroscopy

Spleen: no abnormalities on macroscopy

Stomach and bowel segments: no abnormalities on macroscopy

Kidneys: no abnormalities on macroscopy

**Microscopical examination**:

**Lungs**: Different branches of the bronchial tree show no abnormalities: no inflammation, no malignancy. The lung alveolar parenchyma show normal aeration and no inflammation, no malignancy present.

**Heart**: normal architecture, no inflammation, no malignancy

**Liver**: normal architecture, portal tracts contain bile duct, artery and vein. About half of the portal tracts contain a lymphoplasmocytic inflammatory infiltrate, sometimes forming a lymphoid aggregate. The parenchyma does not show cell drop-out, no centrolobular induction, no inflammation. No malignancy. Overall this is the picture of mild portal hepatitis.

**Kidneys**: Normal cortical and medullar architecture, glomeruli show no abnormalities, different segments of the tubules show no signs of necrosis, inflammation or fibrosis. No interstitial lesions. No vascular lesions.

**Spleen**: normal architecture and presence of red and white pulpa, no inflammation, no bleeding. No malignancy, signs of haematopoiesis

**Tumour**: solid growing tumour consisting of large cells with atypical nuclei.

**Toxicology Report of mouse EX-7706**

**Gross pathology**:

Following median thoracal and abdominal section, following organs were visualized and biopsies were prelevated:

Lungs: no abnormalities on macroscopy

Heart: no abnormalities on macroscopy

Thymus: no abnormalities on macroscopy

Thoracic cavity: no abnormalities on macroscopy, no fluid

Liver: no abnormalities on macroscopy

Pancreas: no abnormalities on macroscopy

Spleen: no abnormalities on macroscopy

Stomach and bowel segments: no abnormalities on macroscopy

Kidneys: no abnormalities on macroscopy

**Microscopical examination**:

**Lungs**: Different branches of the bronchial tree show no abnormalities: no inflammation, no malignancy. The lung alveolar parenchyma show normal aeration and no inflammation, no malignancy present.

**Heart**: normal architecture, no inflammation, no malignancy

**Liver**: normal architecture, portal tracts contain bile duct, artery and vein. About half of the portal tracts contain a lymphoplasmocytic inflammatory infiltrate. The parenchyma does not show cell drop-out, no centrolobular induction, no inflammation. No malignancy. Overall this is the picture of mild portal hepatitis.

**Kidneys**: Normal cortical and medullar architecture, glomeruli show no abnormalities, different segments of the tubules show no signs of necrosis, inflammation or fibrosis. No interstitial lesions. No vascular lesions.

**Spleen**: normal architecture and presence of red and white pulpa, no inflammation, no bleeding. No malignancy, signs of haematopoiesis

**Tumour**: solid growing tumour consisting of large cells with atypical nuclei.

**Toxicology Report of mouse EX-7713**

**Gross pathology**:

Following median thoracal and abdominal section, following organs were visualized and biopsies were prelevated:

Lungs: no abnormalities on macroscopy

Heart: no abnormalities on macroscopy

Thymus: no abnormalities on macroscopy

Thoracic cavity: no abnormalities on macroscopy, no fluid

Liver: no abnormalities on macroscopy

Pancreas: no abnormalities on macroscopy

Spleen: no abnormalities on macroscopy

Stomach and bowel segments: no abnormalities on macroscopy

Kidneys: no abnormalities on macroscopy

**Microscopical examination**:

**Lungs**: Different branches of the bronchial tree show no abnormalities: no inflammation, no malignancy. The lung alveolar parenchyma show normal aeration and no inflammation, no malignancy present.

**Heart**: normal architecture, no inflammation, no malignancy

**Liver**: normal architecture, portal tracts contain bile duct, artery and vein. About half of the portal tracts contain a lymphoplasmocytic inflammatory infiltrate, sometimes forming a lymphoid aggregate. The parenchyma does not show cell drop-out, no centrolobular induction, no inflammation. No malignancy. Overall this is the picture of mild portal hepatitis.

**Kidneys**: Normal cortical and medullar architecture, glomeruli show no abnormalities, different segments of the tubules show no signs of necrosis, inflammation or fibrosis. No interstitial lesions. No vascular lesions.

**Spleen**: normal architecture and presence of red and white pulpa, no inflammation, no bleeding. No malignancy, signs of haematopoiesis

**Tumour**: solid growing tumour consisting of large cells with atypical nuclei. There is tumour necrosis.
