# Supplementary material for: Pharmacological induction of membrane lipid poly-unsaturation sensitizes melanoma to ROS inducers and overcomes acquired resistance to targeted therapy
Source: J Exp Clin Cancer Res. 2023 Apr 19;42:92. doi: 10.1186/s13046-023-02664-7 (PMC10114329; doi:10.1186/s13046-023-02664-7)
Supplement: Supplementary file 3 — Additional file 3. Unedited Western Blots [file 13046_2023_2664_MOESM3_ESM.pptx]

## Slide 1
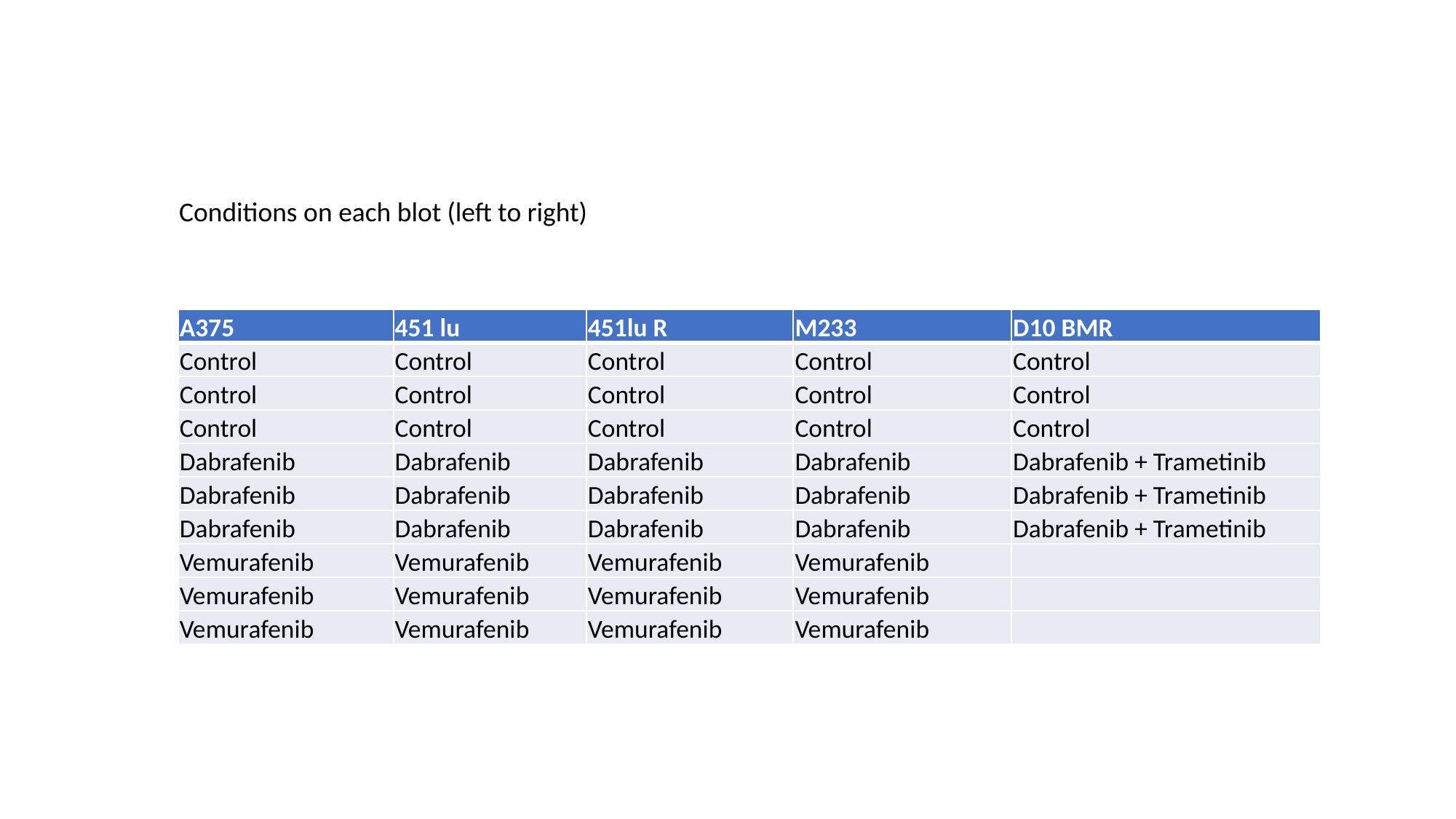

Conditions on each blot (left to right)
| A375 | 451 lu | 451lu R | M233 | D10 BMR |
| --- | --- | --- | --- | --- |
| Control | Control | Control | Control | Control |
| Control | Control | Control | Control | Control |
| Control | Control | Control | Control | Control |
| Dabrafenib | Dabrafenib | Dabrafenib | Dabrafenib | Dabrafenib + Trametinib |
| Dabrafenib | Dabrafenib | Dabrafenib | Dabrafenib | Dabrafenib + Trametinib |
| Dabrafenib | Dabrafenib | Dabrafenib | Dabrafenib | Dabrafenib + Trametinib |
| Vemurafenib | Vemurafenib | Vemurafenib | Vemurafenib | |
| Vemurafenib | Vemurafenib | Vemurafenib | Vemurafenib | |
| Vemurafenib | Vemurafenib | Vemurafenib | Vemurafenib | |

## Slide 2
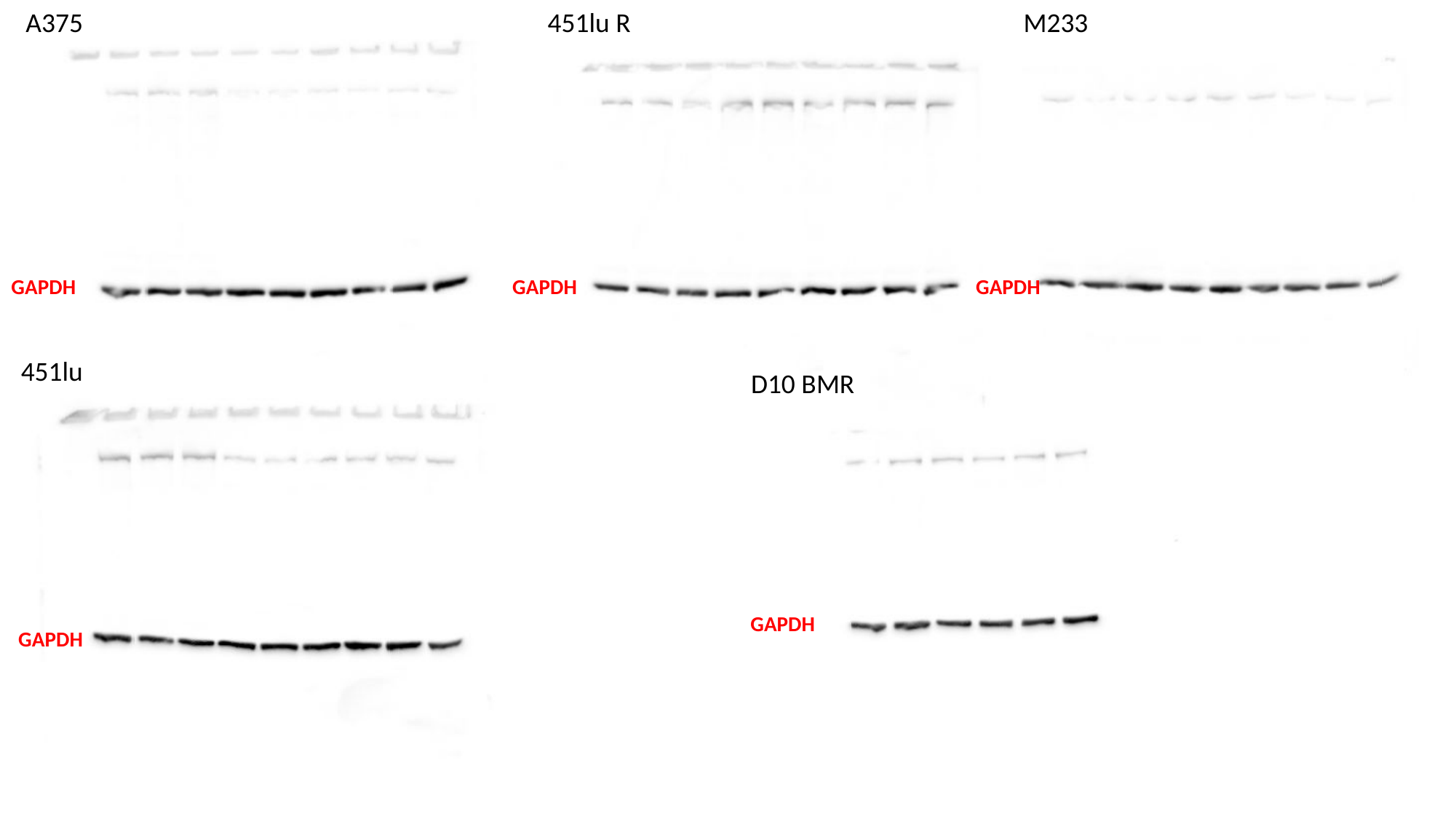

A375
451lu R
M233
GAPDH
GAPDH
GAPDH
451lu
D10 BMR
GAPDH
GAPDH

## Slide 3
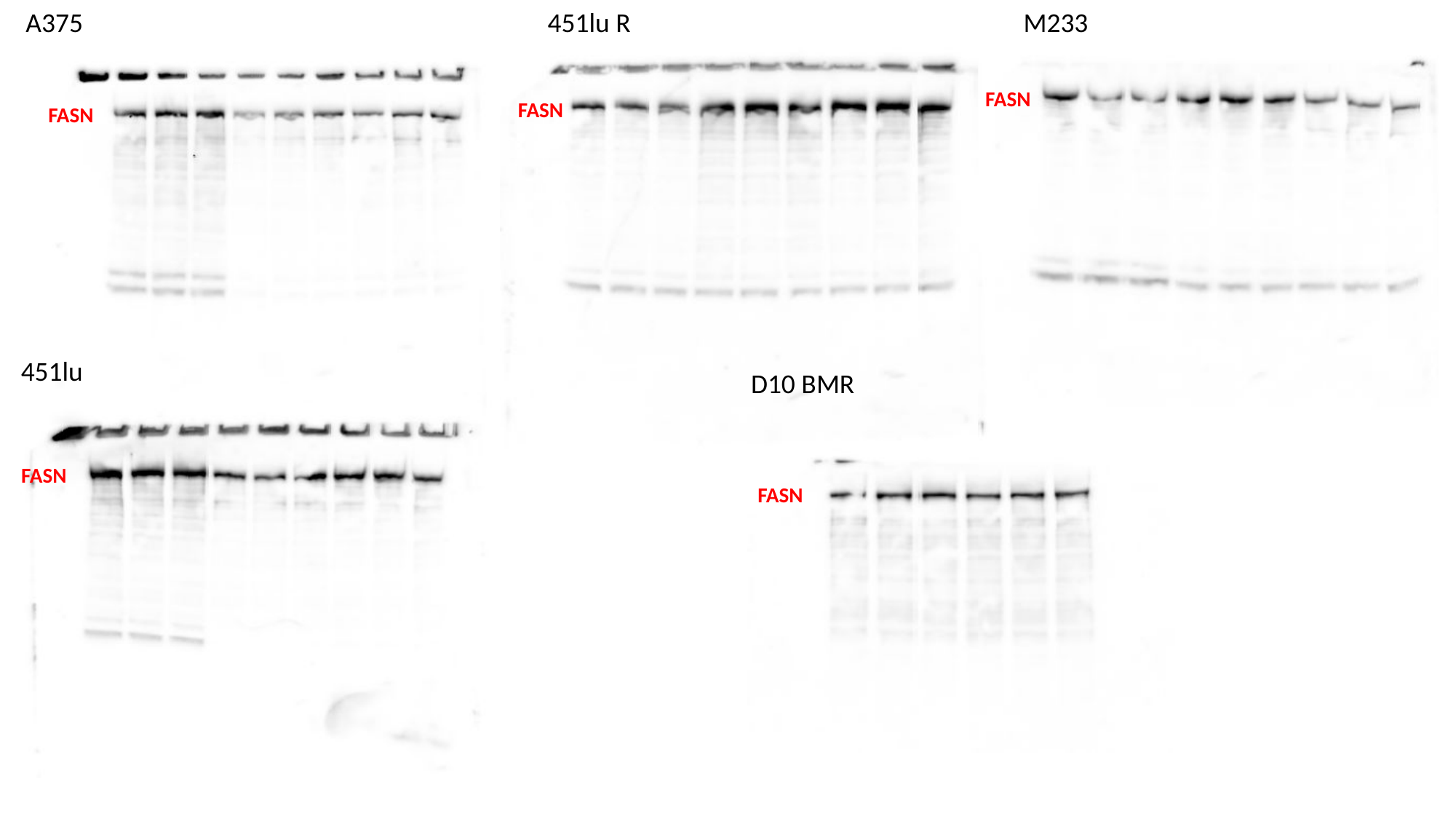

A375
451lu R
M233
FASN
FASN
FASN
451lu
D10 BMR
FASN
FASN

## Slide 4
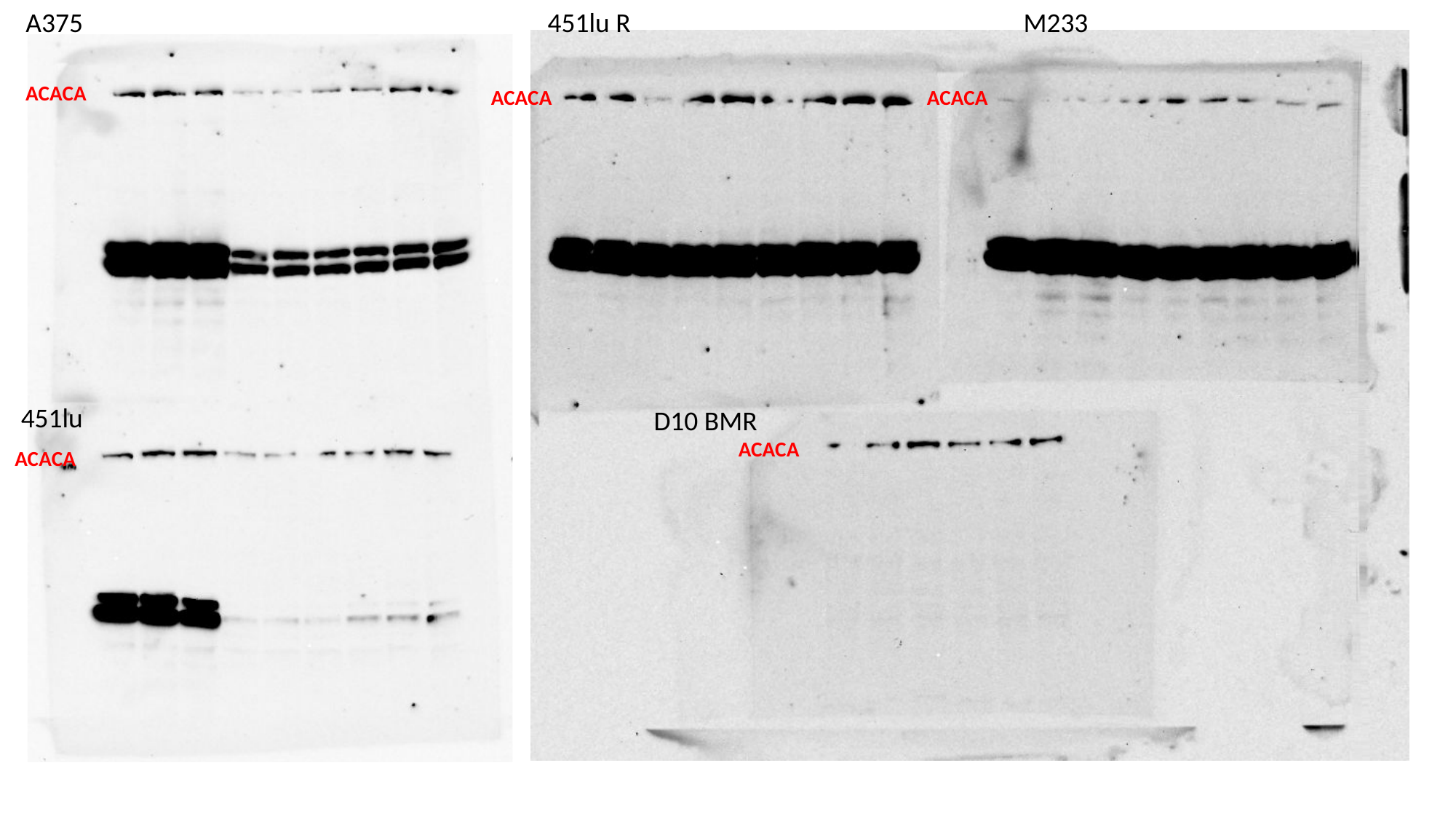

A375
451lu R
M233
ACACA
ACACA
ACACA
451lu
D10 BMR
ACACA
ACACA

## Slide 5
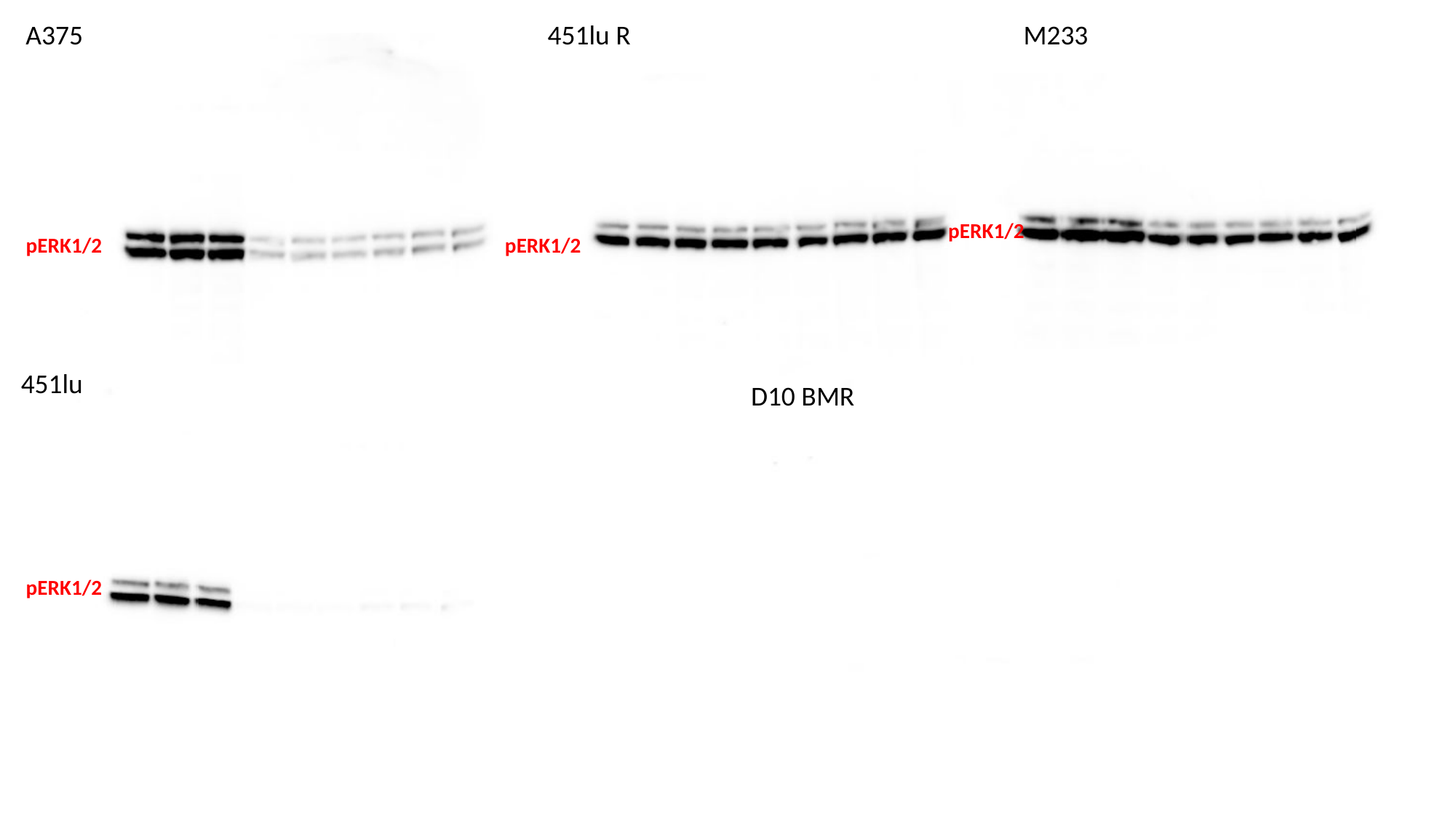

A375
451lu R
M233
pERK1/2
pERK1/2
pERK1/2
451lu
D10 BMR
pERK1/2

## Slide 6
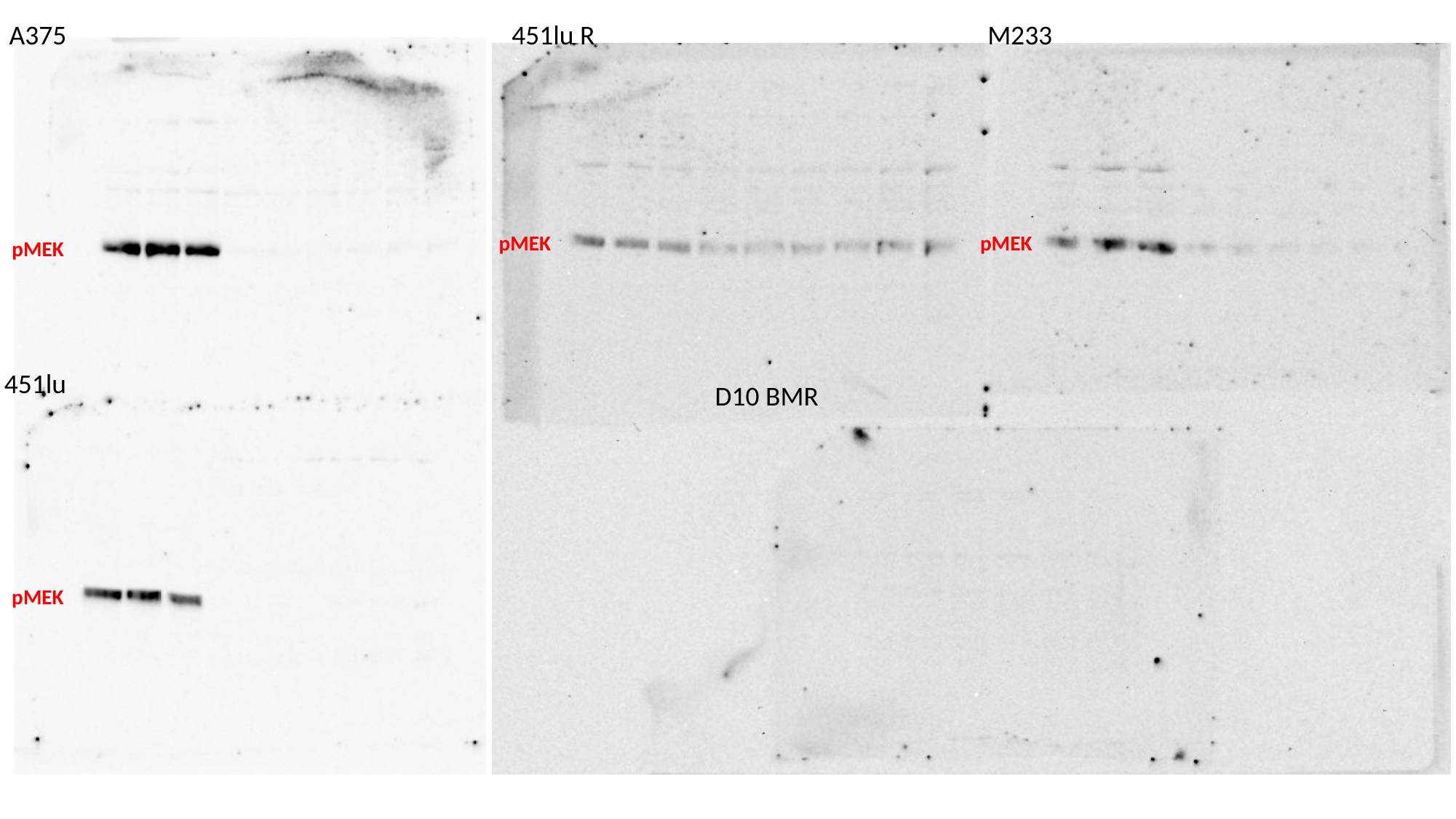

A375
451lu R
M233
pMEK
pMEK
pMEK
451lu
D10 BMR
pMEK
